# MicroRNA-21-Enriched Exosomes as Epigenetic Regulators in Melanomagenesis and Melanoma Progression: The Impact of Western Lifestyle Factors

**DOI:** 10.3390/cancers12082111

**Published:** 2020-07-29

**Authors:** Bodo C. Melnik, Swen Malte John, Pedro Carrera-Bastos, Gerd Schmitz

**Affiliations:** 1 Department of Dermatology, Environmental Medicine and Health Theory, University of Osnabrück, Am Finkenhügel 7a, D-49076 Osnabrück, Germany; johnderm@uos.de; 2 Institute for Interdisciplinary Dermatological Prevention and Rehabilitation (iDerm) at the University of Osnabrück, Am Finkenhügel 7a, D-49076 Osnabrück, Germany; 3 Lower-Saxonian Institute of Occupational Dermatology (NIB), Am Finkenhügel 7a, D-49076 Osnabrück, Germany; 4 Department of Clinical Sciences, Lund University, Jan Waldenströms gata 35, CRC, hus 28 plan 11, 205 02 Malmö, Sweden; pmcbastos@gmail.com; 5 Institute for Clinical Chemistry and Laboratory Medicine, University Hospital, Regensburg, University of Regensburg, Franz-Josef-Strauß-Allee 11, D-93053 Regensburg, Germany; gerd.schmitz@ukr.de

**Keywords:** environment, epigenetics, exosome, melanoma, metabolic syndrome, microRNA-21, prevention, obesity, radiation, therapy

## Abstract

DNA mutation-induced activation of RAS-BRAF-MEK-ERK signaling associated with intermittent or chronic ultraviolet (UV) irradiation cannot exclusively explain the excessive increase of malignant melanoma (MM) incidence since the 1950s. Malignant conversion of a melanocyte to an MM cell and metastatic MM is associated with a steady increase in microRNA-21 (miR-21). At the epigenetic level, miR-21 inhibits key tumor suppressors of the RAS-BRAF signaling pathway enhancing proliferation and MM progression. Increased MM cell levels of miR-21 either result from endogenous upregulation of melanocytic miR-21 expression or by uptake of miR-21-enriched exogenous exosomes. Based on epidemiological data and translational evidence, this review provides deeper insights into environmentally and metabolically induced exosomal miR-21 trafficking beyond UV-irradiation in melanomagenesis and MM progression. Sources of miR-21-enriched exosomes include UV-irradiated keratinocytes, adipocyte-derived exosomes in obesity, airway epithelium-derived exosomes generated by smoking and pollution, diet-related exosomes and inflammation-induced exosomes, which may synergistically increase the exosomal miR-21 burden of the melanocyte, the transformed MM cell and its tumor environment. Several therapeutic agents that suppress MM cell growth and proliferation attenuate miR-21 expression. These include miR-21 antagonists, metformin, kinase inhibitors, beta-blockers, vitamin D, and plant-derived bioactive compounds, which may represent new options for the prevention and treatment of MM.

## 1. Introduction

Starting from a trend-break in 1955, the incidence of malignant melanoma (MM) has increased steadily in Caucasian populations, which points to changes in lifestyle and environment [1,2,3,4]. To clarify the pathogenesis of MM, efforts have been focused on the identification of MM oncogenes, such as *NRAS, BRAF, PTEN, MITF, NEDD9, hTERT* and *KIT*, which have led to an improved understanding of MM etiology and opened new avenues for personalized treatment [5,6,7,8,9,10]. However, not only genetic deviations promote MM initiation, proliferation and progression but also epigenetic mechanisms including aberrant DNA- methylations and changes in microRNA (miR) expression, extensively reviewed elsewhere [11,12,13,14,15,16,17,18,19,20,21,22,23]. The present review focuses on miR-21, which is a key oncogenic miR overexpressed in MM, glioblastoma and other common cancers of Western societies [24,25,26,27]. MiR-21 is also increased in serum and plasma of MM patients and is regarded as a potential biomarker of MM [28,29,30]. This review highlights three major aspects: (1) The role of miR-21 signaling in MM pathogenesis and progression, (2) the impact of environmental factors enhancing miR-21 in MM and MM microenvironment with special attention to exosome-derived miR-21, and (3) potential therapeutic options that attenuate miR-21 signaling in MM. Translational evidence indicates that metabolic, environmental and lifestyle factors increase miR-21 expression including exosomal miR-21 trafficking involved in melanomagenesis and MM progression.

## 2. Methodological Approach

The PubMed database was searched from July 2010 to July 2020 for microRNA-21-melanoma interactions including cutaneous and uveal melanoma, rodent melanoma models, and melanoma cell lines including human A375 and murine B16 melanoma cells. MiR-21 target gene interactions were controlled by TargetScanHuman and miRBase. Selected key words for literature search were: melanoma, cutaneous melanoma, uveal melanoma, malignant melanoma, microRNA-21, miRNA-21, miR-21, radiation, cosmic radiation, electromagnetic radiation, ultraviolet radiation, exosomes, melanoma exosomes, aging, lifestyle, diabetes mellitus, obesity, diabesity, metabolism, metabolic syndrome, melanoma microenvironment, immune regulation, Western diet, hyperglycemic diet, and high-fat diet.

## 3. Mechanism of Action of MiR-21 in Melanoma and Melanoma Cells

### 3.1. MiR-21 in Melanoma Pathogenesis and Progression

MiR-21 targets key genes involved in melanomagenesis and MM progression. MiR-21 regulates genes that are involved in MM proliferation, G_1_/S transition and invasion [31,32]. Its expression steadily raises with the progression of benign nevi to primary and metastatic MM, correlates with Breslow tumor thickness and advanced clinical stage [31,33]. Compared with benign nevi, primary cutaneous MM had an 8.6-fold overexpression of miR-21, which was associated with mitotic activity [34]. Positive sentinel lymph node biopsy (SLNB) was related to increased miR-21 expression in the primary lesion compared with lesions with a negative SLNB (Table 1) [34]. MiRNA-21 is also upregulated in uveal melanoma [35]. Patients with high miR-21 expression show shorter five-year disease-free or overall survival than those with low miR-21 expression [33]. In contrast, antisense-mediated miR-21 inhibition suppresses growth, increases apoptosis and enhances chemo- or radiosensitivity of human MM cells [33]. Thus, miR-21 is a pivotal MM oncomiR that promotes melanomagenesis and MM progression.

### 3.2. MiR-21 Targets in Melanoma Cells

**Figure 1:**
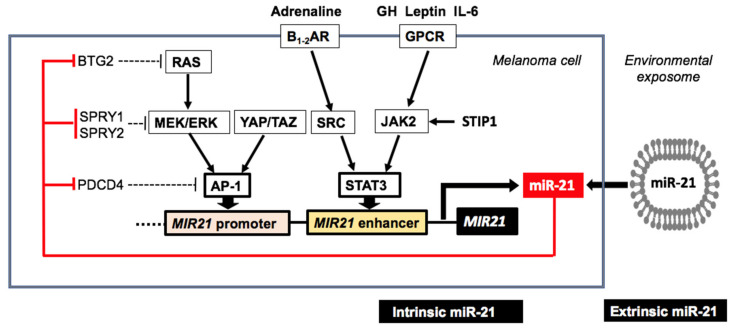
Proposed model showing endogenous and extrinsic miR-21 expression in malignant melanoma (MM). RAS-MEK-ERK-AP-1 signaling as well as signal transducer and activator of transcription 3 (STAT3) signaling increases intracellular miR-21 expression. Yes-associated protein (YAP) and its homolog transcriptional coactivator with PDZ-binding motif (TAZ) increases FOS transcription activating AP-1 composed of FOS and JUN. Endogenous signals such as adrenaline via β-adrenergic receptors (B_1-2_AR) and endocrine hormones such as growth hormone (GH), obesity-induced leptin and inflammation-associated interleukin 6 (IL-6) upregulate STAT3, the enhancer of *MIR21*. Cellular miR-21 levels may be further increased by extrinsic exosome-derived miR-21 derived from various components of the MM environment. MiR-21 via suppression of B-cell translocation gene 2 (*BTG2*), Sprouty 1 (*SPRY1*), Sprouty 2 (*SPRY2*) and programmed cell death 4 (*PDCD4*) potentiates oncogenic RAS-MEK-ERK-AP-1 signal transduction in MM.

MiR-21 expression in MM is inversely associated with nuclear expression of phosphatase and tensin homolog (PTEN) [39]. In human melanoma A375 cells, miR-21 promotes proliferation, migration, and suppresses apoptosis by inhibiting Sprouty 1 (SPRY1), programmed cell death 4 (PDCD4), PTEN and cyclin-dependent kinase inhibitor 2C (CDKN2C) [32,39,40]. Increased cellular miR-21 levels distinguish MM from nevi and correlate with MM cellularity [36]. Remarkably, BRAF or NRAS mutations in MM had no significant effect on miR-21 expression [39]. MiR-21 directly targets Sprouty 1 (*SPRY1*), Sprouty 2 (*SPRY2*), B-cell translocation gene 2 (*BTG2*) and inactivates tumor necrosis factor-α-induced protein 8-like 2 (*TIPE2*), which are key post-transcriptional inhibitors of RAS and RAF, respectively [40,41,42,43,44,45,46,47,48]. Via targeting various inhibitors of the RAS-MEK-ERK pathway, miR-21 may induce an autoregulatory mechanism promoting RAS transformation (Figure 1) [47,48]. MiR-21-mediated suppression of the RAS inhibitor BTG2 enhances RAS-MEK-ERK-AP-1 signaling to maintain enhanced miR-21 expression (Figure 1) [47,48].

### 3.3. Endogenous Upregulation of MiR-21 in Melanoma

*MiR-21* gene expression is primarily upregulated by the enhancer *signal transducer and activator of transcription 3* (STAT3) and the promoter *activator protein 1* (AP-1). At the post-transcriptional level, cellular miR-21 concentrations are also regulated by long non-coding RNAs (lncRNAs). Table 2 presents an overview of melanoma-related miR-21 target genes that are involved in the pathogenesis of MM.

#### 3.3.1. Signal Transducer and Activator of Transcription 3

**Figure 2:**
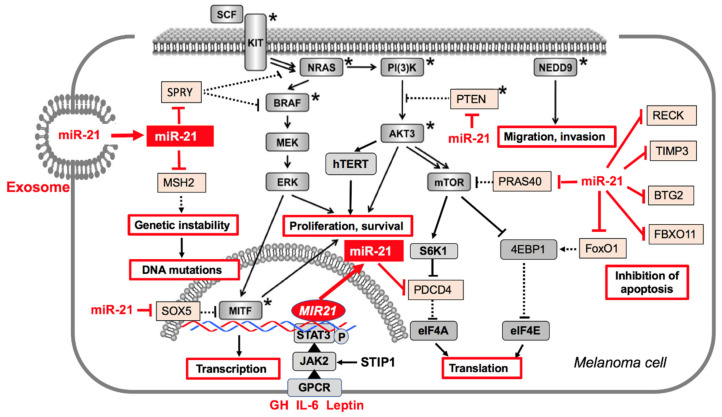
Cell signaling pathways that undergo oncogenic dysregulation in melanoma (MM). Somatic gene mutations in MM, which include *KIT, NRAS, BRAF, MITF, PI3K, PTEN, AKT3*, and *NEDD9* (marked with asterisks), accelerate MM cell proliferation, survival, migration and invasion. In a similar manner, miR-21 promotes these oncogenic signaling pathways by downregulation of various tumor suppressors (*SPRY, BTG2, PTEN, PDCD4*) and inhibitors of mTORC1 and matrix metalloproteinases. MiR-21 inhibits apoptosis and cell cycle control via suppression of *BTG2, FBXO11*, and *FoxO1*. Via suppression of *SOX5*, miR-21 enhances the expression MITF. MiR-21 also attenuates MSH2 activity, thereby compromising DNA mismatch repair resulting in increased genetic instability. Cellular levels of miR-21 either increase by enhanced JAK2-STAT3 signaling or by exosomal transfer of miR-21 by cells of the MM microenvironment. Both somatic mutations in MM and epigenetic modifications of miR-21 have synergistical impacts on oncogenic signaling in MM.

Activation of miR-21 expression via STAT3 represents an epigenetic switch linking inflammation to cancer [61]. STAT3 is commonly activated during MM progression and promotes metastasis [62,63]. Stress-induced phosphoprotein 1 (STIP1) is overexpressed in MM compared to benign nevi and normal skin, respectively [64]. STIP1 stimulates the expression of Janus kinase 2 (JAK2), which activates STAT3 (Figure 1) [64]. STAT3 plays an important role in self-renewal of MM stem-like cells [65]. Accordingly, targeting STAT3 sensitizes human MM cells to the BRAF inhibitor vemurafenib [66]. Intradermal delivery of STAT3 siRNA effectively suppresses MM [67]. A significant association between STAT3 inhibition and the response to nilotinib has also been reported in KIT-mutated MM [68]. STAT3 is an enhancer of miR-21 expression confirmed in B16 MM cells (Figure 1) [57,69,70,71]. Of interest, growth hormone (GH), epidermal growth factor (EGF), leptin, interleukins 6 (IL-6) and IL-10 activate the JAK2-STAT3 pathway thereby promoting miR-21-driven melanomagenesis (Figure 2) [63].

#### 3.3.2. Hippo Pathway

Yes-associated protein (YAP) and its homolog transcriptional coactivator with PDZ-binding motif (TAZ) are key effectors of the Hippo pathway that control cell growth, organ size and tumorigenesis [72]. YAP expression is elevated in most benign nevi and primary cutaneous MMs and uveal MMs [73,74,75]. Gains of copy numbers directly affect YAP and are in the range of 4–10%, and 62% of MMs had copy number alterations affecting Hippo pathway genes [76]. Upon activation, YAP/TAZ-complexes translocate into the nucleus to promote proliferation. An early downstream effector of the Hippo pathway is FOS, a component of AP-1 [72], which promotes miR-21 expression.

#### 3.3.3. Long Non-Coding RNAs

LncRNAs represent a group of transcripts with a length of >200 nucleotides, which play a role in the onset and development of MM [77]. The *X-inactive-specific transcript* (XIST) is one of the first lncRNAs discovered in mammals and plays an essential role in X chromosome inactivation. XIST is dysregulated and acts as an oncogene or as a tumor suppressor in different human malignancies [78]. XIST operates as a miR sponge inhibiting miR-21 [79]. A recent study showed that XIST is overexpressed in MM tissues and cell lines, whereas XIST knockdown inhibits proliferation and migration in MM cells and increases the oxaliplatin sensitivity of resistant MM cells [80]. XIST expression is also upregulated in glioma and glioblastoma stem cells [81,82]. Upregulation of XIST in MM and glioblastoma may represent a counter regulatory mechanism balancing enhanced miR-21 expression.

Downregulated expression of the tumor suppressor lncRNA *maternally expressed gene 3* (MEG3) promotes MM growth and metastasis [58]. Thereby, lncRNA MEG3 functions as a sponge of miR-21 [58]. Downregulated lncRNA MEG3 increased miR-21 associated with suppressed expression of E-cadherin, a newly recognized epigenetic target of miR-21 [58]. Notably, increased expression of lncRNA MEG3 has also been reported in gliomas [83].

LncRNA *growth arrest-specific transcript 5* (GAS5) inhibits the migration and invasion of MM cells [84]. Expression of GAS5 is downregulated in MM tissues compared to adjacent normal tissues [85]. Lentiviral-mediated overexpression of lncRNA GAS5 reduces invasion activity in human MM cells [86]. Remarkably, lncRNA GAS5 functions as a sponge of miR-21 [87,88]. In a feedback loop, miR-21 suppresses the expression of lncRNA GAS5 [89]. There is an emerging interest in exosome-derived non-coding RNAs in cancer biology [90,91], which might also have an impact on melanomagenesis. Taken together, lncRNAs are important epigenetic regulators of miR-21 expression (Table 3).

## 4. Exosome-Mediated MiR-21 Transport

### 4.1. Exosome-Mediated Transfer of MiR-21 to Melanocytic Lesions

MiR-21 in MM cells is either synthesized intracellularly or transferred from the tissue environment by miR-21-enriched exosomes that recently gained high interest within the pathogenesis of various skin diseases including MM [96,97]. Exosomes are extracellular vesicles (EVs) of endocytic and secretory exocytic origin that mediate three-dimensional communication between cells. Exosomes transport cellular components such as miRs, mRNAs, lncRNAs, circular RNAs, proteins and DNAs. They are secreted into body fluids by multiple cell types, including keratinocytes, fibroblasts, adipocytes, benign melanocytes and MM cells. MM-derived exosomes contain intact and functional mRNAs, small RNAs (including miR-21), and proteins (PD-L1) that can alter the cellular environment to favor MM growth [98,99].

The melanocyte appears in close vicinity with epidermal keratinocytes, dermal fibroblasts, subcutaneous adipocytes and immune cells that all exchange exosomes. Exosomes are a special class of EVs that represent nanoparticles of 50–180 nm in diameter. They transport increased amounts of miR-21 of bystander cells to melanocytes, especially after cellular stresses such as ultraviolet (UV) irradiation, free radicals or metabolic alterations. Increased exosomal miR-21 traffic to melanocytes may represent a widely ignored molecular mechanism promoting melanomagenesis and MM progression, which will be discussed in more detail.

### 4.2. Melanoma-Derived MiR-21-Enriched Exosomes and Melanoma Progression

Tumor cell-derived exosomes regulate target gene expression in normal cells [100]. Exosomal miR-21 uptake results from clathrin-mediated endocytosis and macropinocytosis [100]. MM cells are involved in intensive exosome traffic. They either perceive exosome signals from their environment as bystander cells or themselves secrete tumor-promoting exosomes into their environment [101,102,103,104]. MM exosomes mediate pro-tumor processes including angiogenesis, immune dysregulation and modification of the tissue microenvironment [104]. Metastatic MM secretes a higher exosome amount than primary MM, and acidic pH increases exosome secretion [105]. MM exosome production, transfer and programming of bone marrow cells support tumor growth and metastasis [106]. MM-derived exosomes induce a reprogramming of fibroblasts into cancer-associated fibroblasts (CAFs) [104,107]. In accordance, glioma cells shape their microenvironment and communicate with the surrounding microglia [108]. Uptake of glioma cell-derived exosomal miR-21 by microglia cells results in their reprogramming to create a favorable microenvironment for glioma progression [108]. Likewise, MM cells modulate their tumor microenvironment by modulating exosome transfer [107,109]. MM-derived exosomes promote epithelial-mesenchymal transition (EMT) in primary melanocytes through paracrine/autocrine signaling in the tumor microenvironment [110].

Accordingly, patients with metastatic sporadic MM exhibit higher plasma exosomal miR-21 levels, when compared to familial MM or unaffected control subjects [111]. Surprisingly, no substantial differences in miR-21 expression were detected between familial MM patients and unaffected controls [111], pointing to a predominant role of miR-21 in sporadic MM, which is under stronger influence of environmental compared to genetic factors. Notwithstanding, copy numbers of plasma miR-21 correlate with tumor burden in MM patients [112]. MiR-21 is positively correlated with the TNM stage and represents an independent risk factor for MM metastasis [37]. Of interest, increased expression of miR-21 has also been detected in vitreal exosomes of patients with uveal melanoma as well as in formalin-fixed, paraffin-embedded uveal melanoma specimens [113].

**Figure 3:**
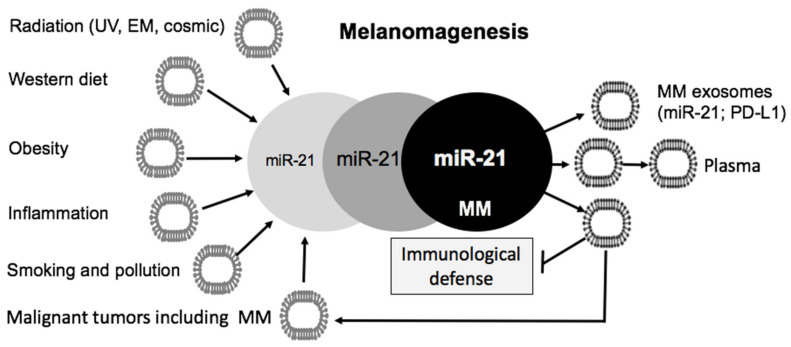
Illustration of exosome-driven melanomagenesis. Environmental and individual factors increase the total burden of miR-21-enriched exosomes. Melanoma (MM) cell levels of miR-21 increase steadily during melanomagenesis. Metastatic MM secretes high amounts of MM-derived miR-21- and PD-L1-enriched exosomes that compromise local and distant tumor defense mechanisms.

MiR-21 promotes tumorigenesis in MM, glioblastoma and prostate cancer via inhibition of pivotal tumor suppressors (Figure 3) [33,40,49,50,72,114,115]. MiR-21 modifies the extracellular matrix by suppressing critical inhibitors of matrix metalloproteinases including TIMP3 and RECK [55,56,116]. In fact, increased expression of miR-21 enhances the invasive potential of MM cells through TIMP3 inhibition [56]. Furthermore, miR-21 induces tumor angiogenesis through targeting PTEN, activating AKT and ERK1/2 signaling, thereby enhancing HIF-1α and VEGF expression as well as by targeting FASLG and angiotensin II [115,117,118,119,120].

### 4.3. Immunological Surveillance

MM can be considered a disease of immune dysfunction with a failure of immune recognition, which is the rationale of immune-checkpoint inhibition (ICI) [121,122]. ICI augments neoantigen-specific CD8+ T cell responses, resulting in tumor regression [123]. Tissue-resident CD8+ memory T cells (TRM) play a vital role for host immune responses to cancer [124]. Of interest, skin-resident memory T cell responses to MM are generated naturally as a result of autoimmune vitiligo [125]. In the epidermis, TRM mediate anti-tumor immunity and promote an MM-immune equilibrium [125,126]. Patients with high intratumoral CD8+ T cells display a higher response to BRAF or BRAF/MEK inhibitors [127]. The dynamic network of miRs is of pivotal importance for the regulation of T cell responses. Intriguingly, upregulation of miR-21 biases the transcriptome of differentiating T cells away from memory T cells and toward inflammatory effector T cells [128]. Such a transcriptome bias is also characteristic of T cell responses in older individuals who have increased miR-21 expression, which is reversed by antagonizing miR-21 [128]. Thus, T cells with high miR-21 expression disfavor the induction of transcription factor networks involved in memory T cell differentiation, which plays a key role in the immunological surveillance of MM.

MiR-21 expression in cells of the tumor immune infiltrate, particularly macrophages, is responsible for promoting tumor growth, whereas the absence of miR-21 in tumor-associated macrophages causes a global rewiring of their transcriptional regulatory network that is skewed toward a pro-inflammatory angiostatic phenotype [129]. This promotes an anti-tumoral immune response characterized by a macrophage-mediated improvement of cytotoxic T-cell responses through the induction of cytokines and chemokines [129]. Moreover, miR-21 contributes to macrophage M2 reprogramming of tumor infiltrating myeloid cells (TIMs) promoting MM metastasis [130]. In contrast, miR-21 deficient B16 mouse melanoma upregulate PD-L1 expression in macrophages, promote macrophage M1 polarization with anti-tumor activity [38]. Of notice, miR-21 suppresses IL12A, the p35 subunit of interleukin 12 (IL-12) [59]. IL-12 exhibits anti-tumor activities via regulation of both innate (natural killer cells) and adaptive (cytotoxic T lymphocytes) immunity [131,132]. Several studies have addressed the use of IL-12 for melanoma therapy due to its immunoregulatory function and anti-tumor activity mediated by stimulation of T and NK effector cells [133,134].

MM exosomes provoke immune suppression and defective dendritic cell (DC) functions [135]. MM exosomes suppress proliferation in CD8+ T cells and downregulate *killer cell lectin-like, subfamily K, member 1* (NKG2D) expression in NK cells [136]. In addition, MM cells secrete PD-L1 through exosomes, which exhibit immunosuppressive activities and inhibit T-cell activation [137]. Furthermore, MM-derived exosomes downregulate T-cell responses through decreased T-cell receptor (TCR) signaling and diminish cytokine and granzyme B secretions [138] (Figure 3).

### 4.4. MiR-21 Overexpression in Melanoma-Related Tumors

MiR-21 is also upregulated in glioblastoma and prostate cancer [93,139,140,141,142], two common cancers of Western societies that are associated with MM [143,144,145]. Increased risk of prostate cancer has been associated with the occurrence of late adolescent acne [146,147]. Of notice, in US women [148], but not in Swedish men [149], teenage acne was associated with increased MM risk. Since acne has been linked to the Western exposome comprising diet, medication, pollutants, psychosocial and other environmental and lifestyle factors [150], it is conceivable that these factors also have an impact on MM via upregulation of miR-21 [151].

## 5. Environmental Factors Upregulating MiR-21

### 5.1. Radiation

Not only ultraviolet (UV) radiation, which is today’s primary focus in melanomagenesis, but also other spectra of electromagnetic radiation enhance miR-21 expression as outlined in more detail below.

#### 5.1.1. Ultraviolet Irradiation

Exposure to UV radiation from sunlight or tanning beds contributes to UV-induced DNA damage, oxidative stress, and inflammation in the skin playing a dominant role in melanomagenesis and DNA mutations causing MM [152,153]. However, key mutations in MM are not UV-signature mutations (C⟶T) including the BRAF^V600E^ mutation found in 60% of MMs and NRAS mutations detected in 15–20% of MMs, respectively [154]. Therefore, UV radiation alone may not explain all mutagenic effects in MMs. Notwithstanding, these non-UV-signature mutations are more common in sun-exposed skin [155,156,157,158]. MMs of the head and neck are associated with chronic patterns of sun exposure, whereas trunk MMs are related to intermittent patterns of sun exposure, supporting the hypothesis that MMs may arise through divergent causal pathways [159].

Intriguingly, UV radiation induces the release of keratinocyte-derived exosomes that communicate with melanocytes to regulate pigmentation [160,161]. EVs released by melanocytes after UV-A irradiation promote intercellular signaling with increased miR-21 expression in keratinocytes [162]. Notably, UV radiation upregulates miR-21 in keratinocytes, fibroblasts and melanocytes [163,164,165,166,167]. MiR-21 enhances the expression of microphthalmia-associated transcription factor (MITF) by targeting SOX5, an inhibitor of MITF [51]. MITF represents a melanocytic lineage-specific transcription factor, which plays a key role in melanomagenesis [168]. Accordingly, SOX5 knockdown upregulates MITF in MM cells [52]. Furthermore, miR-21 controls the DNA mismatch repair (MMR) protein MSH2, which is a crucial caretaker of the MMR including transcription-coupled repair [169]. MMR deficiency is a frequent condition in MM [170]. Reduced or defective expression of MSH2 has been associated with high genomic instability, poor MM prognosis, and metastasis [114,171,172]. Decreased expression or function of MSH2 is either a result of mutation-derived dysfunction of MSH2 or miR-21-mediated downregulation of MSH2 [53,114,171,172,173]. Thus, UV-induced generation of exosomal miR-21 with subsequent uptake of miR-21 by melanocytes may promote genetic instability and gene mutations driving melanomagenesis.

#### 5.1.2. Cosmic Ionizing Irradiation

Airline pilots and cabin crew members have about twice the risk of MM compared to the general population [174,175]. Cosmic radiation primarily consists of neutrons and gamma rays allowing high linear energy transfer (LET) [176], which stimulates miR-21 expression through the STAT3 pathway (Figure 1) [177,178]. Ionizing radiation-induced miR-21 promotes EMT and angiogenesis by downregulation of PTEN [115,179]. Notably, ionizing radiation causes bystander effects on neighboring non-irradiated cells via transfer of exosomes enriched in miR-21 [180,181]. Cosmic radiation may thus upregulate miR-21 levels of epidermal keratinocytes and bystander melanocytes, a potential contribution to melanomagenesis in aircrew members.

#### 5.1.3. Electromagnetic Radiation

Aircrew members are also exposed to electromagnetic fields [182]. Magnetic field levels in the cockpit have a mean value of approximately 17 milliGauss, while cabin measurements are lower [182]. Remarkably, pulsed electromagnetic fields enhance miR-21 expression in human bone marrow stromal cells [183]. Thus, pilots are exposed to a wide spectrum of miR-21-inducing radiation including cosmic ionizing radiation, UV-A radiation (passing through cockpit windshields [184]) and electromagnetic fields in the cockpit including radio transmission.

A correlation between frequency modulation radio transmitter density and MM incidence has been reported in a study involving 23 European countries [185]. Of notice, pilots also have a higher risk for glioma [186,187]. An association of MM and glioma risk has also been observed in the general population [143], which may be linked to their common progenitors of neural crest-derived glia cells and melanoblasts [188]. Recently, an association between mobile phone use and low-grade glioma has been reported [189,190], whereas a cohort study in Denmark found no significant association between mobile phone use (another source of electromagnetic radiation) and MM [191]. Thus, translational evidence suggests that not only UV, but a much wider spectrum of electromagnetic radiation upregulates the expression of miR-21 that may affect MM and glioma development via exosomal miR-21 transfer.

### 5.2. Metabolic Deviations Upregulating MiR-21

#### 5.2.1. Metabolic Syndrome and Melanoma Risk

An important contributor to the pandemic of cardiovascular disease is overweight, obesity, insulin resistance, type 2 diabetes mellitus (diabesity), and arterial hypertension, major components of the metabolic syndrome [192,193]. Diabesity is associated with changes in the maternal environment, which can affect developmental processes [194]. Recently, glioma progression has been related to diabesity [195]. There is compelling epidemiological evidence that increased birth weight (BW) is associated with an increased risk of obesity and type 2 diabetes mellitus (T2DM) [196,197,198]. Notably, high BW (>4000 g) was associated with increased risk of obesity [196]. Especially, early development of obesity predicted obesity in adulthood, predominantly for children who were severely obese [199]. In the United States, the 2017 incidence of fetal macrosomia, defined as BW >4000 g, was 8.07% [200]. In a murine model, fetal macrosomia has been identified as an independent risk factor of the metabolic syndrome [201]. Intriguingly, fetal and childhood growth trajectories are not only linked to an increased risk of the metabolic syndrome but also of MM as outlined below.

#### 5.2.2. Birth Weight and Height in Childhood

A case-control study of 1396 cases of MM diagnosed before the age of 30 in 1988–2013 and 27,920 controls in California demonstrated that high BW (>4000 g) compared to normal BW is associated with a 19% higher risk for MM before the age of 30 [202]. In placental tissue of macrosomic babies, increased levels of miR-21 have been detected [203,204]. Placental-derived exosomes and their miR cargo are related to pregnancy complications [205,206] and may also have an impact on melanomagenesis during the fetal growth period.

Height at ages 7–13 years can also affect MM risk, according to data from the Copenhagen School Health Records Register [207]. A positive association between genetically-predicted height and MM risk has also been observed [208]. Of relevance, cow’s milk consumption during childhood has been associated with increased linear growth [209]. Exosome transfer of bovine milk miR-21 [210], which is identical to human miR-21, increases mTORC1 signaling [211,212,213,214,215,216,217,218,219]. Activated mTORC1 promotes osteogenesis and myogenesis [216,217] and plays a key role in acne and BRAF^V600E^-related MM [214,215,218]. Thus, accelerated growth in the fetal period and during childhood is related to excessive miR-21/mTORC1 signaling that may also affect melanomagenesis.

#### 5.2.3. Overweight and Obesity

Recent studies support a link between obesity and MM occurrence and progression [220,221,222,223,224,225]. Studies with the 3T3-L1 adipocyte cell line as well as ex vivo subcutaneous and visceral adipose tissue conditioned medium have shown that adipocyte-released factors increase MM cell overall survival [226]. Adipocyte-derived conditioned media activate AKT and mTORC1 in MM cells and stimulate proliferation, migration, and invasion [227]. In addition, adipocyte-derived exosomes promote MM aggressiveness by increasing fatty acid oxidation [228,229]. Interestingly, high-fat diet (HFD)-induced obesity in mice increases miR-21 content of white adipose tissue and upregulates the proliferation of human adipose tissue-derived mesenchymal stem cells [230,231]. Exosomes released from adipocyte-derived stem cells exhibit elevated miR-21 levels that induce angiogenesis through AKT and ERK activation and enhance the migration and proliferation of HaCaT cells [232,233]. Moreover, the adipokine leptin promotes MM growth and activates STAT3 [220,234,235], which induces miR-21 expression (Figure 1) (Table 3) [229,235]. In mature human adipocytes, miR-21 is also upregulated by tumor necrosis factor-α (TNF-α), IL-6, resistin and free fatty acids (FFAs) [236]. Apparently, adipocytes of obese individuals secrete miR-21-enriched exosomes into the microenvironment of melanocytes and into the systemic circulation (Figure 3). Cancer-associated adipocytes (CAAs) release FFAs, which are transferred to cancer cells and are used for energy production through β-oxidation [237]. As FFAs induce the expression of miR-21 [236], upregulation of miR-21 in CAA-derived exosomes is expected. In fact, peritumoral CAAs isolated from the omental adipose tissue surrounding metastatic ovarian cancer secrete miR-21-enriched exosomes, which are transferred to cancer cells [238]. Thus, epigenetic evidence indicates that exosomal transfer of miR-21 by adipocytes of obese individuals and CAAs may promote melanomagenesis.

#### 5.2.4. Diabetes Mellitus

A recent meta-analysis reported an increased risk of MM in patients with T2DM [239]. Moreover, miR-21 is increased in the blood of T2DM patients [240,241,242]. In a diabetic mouse model, the curcumin analog C66 inhibits diabetes-related induction of miR-21 in analogy to miR-21 reductions by locked nucleic acid-modified anti-miR-21 (LNA-21) [243]. Thus, overexpressed miR-21 in T2DM might represent a molecular link between T2DM and MM.

In type 1 diabetes mellitus (T1DM), 𝛽-cell-derived exosomal miR-21 cargo significantly increased in response to inflammatory cytokines [244], therefore T1DM might also be implicated in melanomagenesis, especially in the context of chronic inflammation.

#### 5.2.5. Arterial Hypertension

An association between arterial hypertension and MM risk has been reported [245,246,247]. The mechanisms of this interaction are yet unknown. A potential link is the recent observation of increased circulatory levels of miR-21 in patients with hypertensive heart disease [92]. Suppression of miR-21 prevents hypertrophic stimulation-induced cardiac remodeling by regulating PDCD4, AP-1, and TGF-β1 signaling pathways [92].

#### 5.2.6. Western Diet

Western diet might may also drive melanomagenesis [151,248]. For instance, the chronic consumption of hyperpalatable processed foods high in sugar, fat, salt, and flavor additives can lead to excessive energy intake and obesity [249]. It has been demonstrated in mouse models, that diet-induced obesity directly increases MM initiation and progression [220,250]. Furthermore, a high intake of sugars, mostly sucrose, glucose and fructose, has been identified as a potential risk factor of MM in a recent Italian study [251]. An overall high glycemic load (GL) has been associated with increased risk of MM and acne [252,253]. Of notice, high GL diets might overactivate mTORC1, particularly under the context of positive energy balance [203,254]. In response to high glucose intake, endothelial cells overexpress miR-21 [255]. In addition, high glucose-stimulated expression of miR-21 inactivates PRAS40, a negative regulator of mTORC1 [60]. Fructose can also increase plasma levels of miR-21 [256].

It has been suggested to replace this unfavorable dietary pattern by low-carbohydrate high-fat ketogenic diets [257,258], but these might negatively impact MM [258,259,260]. For instance, oncogenic BRAF^V600E^ upregulates HMG-CoA lyase, which converts HMG-CoA to acetyl-CoA and the ketone body acetoacetate, that selectively enhances BRAF^V600E^-dependent MEK1 activation in MM [261]. A high-fat ketogenic diet increases serum levels of acetoacetate leading to enhanced growth of BRAF^V600E^-expressing human MM cells in xenograft mice [262]. Notably, BRAF^V600E^ is negatively controlled by members of the Sprouty family of tumor suppressors [262], which are targets of miR-21 [40,42,151]. It is thus conceivable that miR-21-mediated downregulation of Sprouty enhances BRAF^V600E^-driven MM growth (Figure 2).

There is recent interest in exosomes derived from foods [263,264], particularly those delivered by pasteurized milk [212]. Milk consumption might affect MM through activation of mTORC1 signaling due to its amino acid profile and endocrine effects (increase of insulin-like growth factor 1) and by transfer of miR-21-enriched milk exosomes to the milk consumer [210,211,212,213]. Indeed, in mice, orally administered cow’s milk exosomes are bioavailable, distribute in various tissues and organs and affect metabolic regulation [265,266]. Moreover, consumption of milk fat, which is also a rich source of miR-21 [267], enhances telomere length [268]. Interestingly, miR-21 via inhibiting PTEN activates telomerase (hTERT) [269,270], which is a further feature of MM [271,272].

Western diets are also characterized by excessive intake of alcohol [273], which can impact MM [274]. Chronic alcohol intake, especially in combination with HFD results in persistent ketonuria associated with increased serum levels of acetoacetate [275], which accelerates BRAF^V600E^-MEK1 signaling. Furthermore, excessive alcohol consumption combined with acute psychological stress upregulates miR-21 [276,277].

Dietary xenobiotics, present in the Western diet, may also impact MM. For instance, chronic exposure to polychlorinated biphenyls (PCBs), mainly from fatty fish, are associated with a four-fold increased risk of MM [278], perhaps because PCBs increase miR-21 expression [279,280]. Finally, Western diets trigger inflammation [281], which is critically involved in melanomagenesis, as outlined below.

#### 5.2.7. Smoking and Pollution

Smoking has recently been identified in some studies as a predictor of poor MM outcome [282,283], whereas other studies found no or even inverse relations between smoking and MM incidence [284,285,286]. In contrast to these conflicting epidemiological studies, molecular evidence appears to be more consistent. For example, nicotine induces the expression of miR-21 and promotes EMT in esophageal cells [287]. Cigarette smoke induces the release of miR-21-enriched exosomes from bronchial epithelial cells [288]. Exosomes released from nicotine-stimulated macrophages increase miR-21/PTEN-mediated vascular smooth muscle cell migration and proliferation [289].

**Figure 4:**
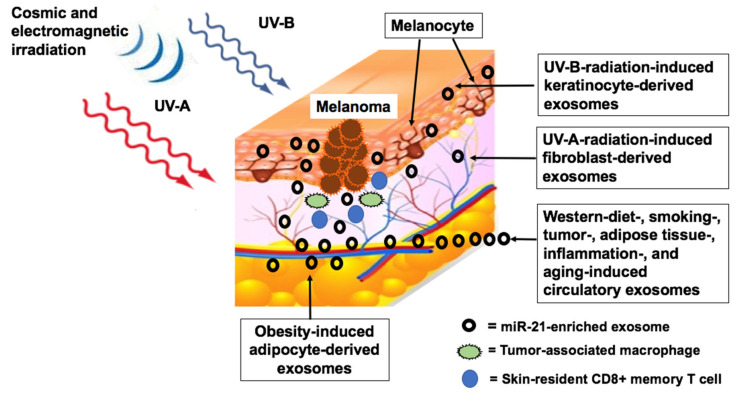
Synoptic model depicting exosomal miR-21 traffic in the melanocyte and melanoma microenvironment. Cosmic and UV irradiation induce the release of keratinocyte-and fibroblast-derived miR-21-enriched exosomes, which may increase melanocyte miR-21 levels. Adipose tissue-derived exosomal miR-21 in obesity may further increase melanocyte miR-21 levels. Melanocyte miR-21 may be further enhanced by circulatory exosomes generated by a Western diet, milk consumption, smoking, inflammation, tumors and aging. Increased miR-21 levels in melanoma-associated macrophages and skin resident CD8+ memory T-cells further promote MM progression.

Pollution may also affect MM. In a study with human bronchial epithelial cells, diesel exhaust particles increased miR-21 expression and activated the PI3K/AKT pathway [290]. Possibly, smoke- and pollution-induced airway epithelial cell-derived exosomes may enter the systemic circulation promoting melanomagenesis (Figure 4).

### 5.3. Hormonal Factors

#### 5.3.1. Androgens

Accumulating epidemiological evidence associates prostate cancer, acne and MM [144,145,148,291], with androgen-dependence as a possible link [291]. Indeed, the promotor of *MIR21* is upregulated by androgen receptor (AR) (Table 3) [93]. Notably, patients with AR-positive MM have worse survival outcomes compared to patients with AR-negative MM [292]. AR also promotes MM metastasis via MITF signaling [292]. In accordance, dehydroepiandrosterone (DHEA), often administered for anti-aging purposes [293], also upregulates the transcription of miR-21 [294].

#### 5.3.2. Growth Hormone

Human metastatic MM cell lines express high levels of growth hormone receptor (GHR) and respond to GH with increased proliferation [295]. GH promotes, while GHR knockdown attenuates MM progression [296]. GH affects multiple oncogenic signaling pathways, especially JAK2-STAT3 [296]. The GH-GHR axis induces chemoresistance in human MM by driving MITF-regulated and ABC-transporter-mediated drug clearance pathways [297]. Increased GH-JAK2-STAT3 signaling may thus explain the association between GH administration and MM (Figure 2) [298,299,300]. Interestingly, the prepubertal somatotropic axis can be modified by milk consumption [301,302]. Daily milk consumption in 10- to 11-year-old children increases their plasma GH levels and accelerates their longitudinal growth [302]. This is of concern because height at ages 7–13 years significantly increases MM risk [209]. Elevated GH plasma levels have also been observed after ingestion of gelatin protein, soy protein, and α-lactalbumin, a whey protein [303]. Of notice, recombinant GH is frequently abused in combination with androgens and whey protein supplements to gain muscle mass [304], a doping procedure that may increase MM risk.

#### 5.3.3. Vitamin D

Vitamin D (VD) deficiency has been associated with a poorer outcome in MM [282,283], and has been correlated with BRAF-mutated MM [305]. Higher 25-hydroxyvitamin D_3_ (25OHD_3_) levels are associated with lower Breslow thickness at diagnosis and are independently protective of relapse and death of MM [306], whereas patients with low 25OHD_3_ concentrations are associated with greater Breslow thickness and reduced survival [307,308]. Clinicopathological analyses have shown positive correlations between low or undetectable expression of VD receptor (VDR) in MM with accelerated tumor progression [308,309]. Notably, VD upregulates the expression of programmed death ligand 1 (PD-L1) on both epithelial and immune cells, suggesting an interaction with immune checkpoint inhibitors [310]. Intriguingly, VDR inhibits the expression of miR-21 [54,94,95,311,312]. Furthermore, the expression of the miR-processing ribonuclease DICER1 positively correlates with VD metabolite levels [311]. MiR-21 negatively regulates VD production through inhibition of CYP27B1 encoding 25OHD_3_-1-α-hydrolase, which converts 25OHD_3_ to its active form, 1α,25-dihydroxyvitamin D_3_ (1α,25-[OH]_2_D_3_) [54]. The recently observed correlation between BRAF^V600E^ mutation status and VD deficiency may thus be explained by BRAF^V600E^ mutation-induced overexpression of CYP24A1, encoding 24-hydroxylase [313], the mitochondrial enzyme responsible for inactivating VD metabolites through the C-24 oxidation pathway [314]. Compared to normal epidermis, highest mean CYP24A1 levels were found in nevi and early stage MMs [315]. Thus, miR-21 signaling is closely related to VD homeostasis. Interestingly, UV radiation induces the synthesis of various other photoproducts that may have anti-MM activity [316,317,318,319]. This, together with VD, may offer a partial explanation for the puzzling lower incidence of MM in outdoor workers and individuals with higher annual UV exposure [320].

### 5.4. Aging and Chronic Inflammation

#### 5.4.1. Aging

The incidence of MM increases with age [321,322,323]. Whereas MM development in younger patients is the result of genetic factors, particularly related to multiple nevi, in older patients, environmental factors play a predominant role [322]. Higher age leads to worse survival in stages I, II and III [323]. Remarkably, plasma levels of miR-21 increase with age [324,325] and reach highest levels at the age of 66 years [324], coinciding with the climax of MM incidence.

#### 5.4.2. Chronic Inflammation

Certain pro-inflammatory environmental and lifestyle factors promote cancer [281,326] through nuclear factor-κB (NF-κB) and STAT3 signaling pathways [327,328]. Furthermore, miR-21 activates the NLRP3 inflammasome [329,330]. Increased expression of miR-21 and exosomal miR-21 is associated with inflammation in various conditions, such as diabetes mellitus (type 1 and 2), chronic renal fibrosis, and atopic diseases [331,332,333]. Chronic inflammation may thus enhance the systemic burden of exosomal miR-21 that may reach the skin promoting melanomagenesis (Table 4).

## 6. Therapeutic Suppression of MiR-21

### 6.1. Vemurafenib

MiR-21 could be a new target for the prevention and treatment of MM. Currently, the treatment of BRAF^V600^-mutated metastatic melanoma with BRAF inhibitors gives a response rate of about 50% with a progression-free survival in the range of 6–7 months [336]. In vemurafenib-treated metastatic MM patients, a significant decrease in miR-21 expression was observed in BRAF-mutated in comparison with BRAF wild-type patients [336]. Thus, MEK-ERK kinase inhibition in MM may attenuate miR-21 expression. In contrast, vemurafenib significantly increased total RNA and protein content of released EVs of MM cells associated with a significant increase of miR-211, which reduces the sensitivity of MM cells to vemurafenib [337].

### 6.2. Metformin

The antidiabetic drug metformin decreases the risk of various cancers including MM [338]. Metformin reduces MM cell growth in vitro and in rodent models [339,340,341]. Whereas a recent pilot study indicates no benefit of metformin monotherapy in MM [342], improved MM outcomes have been reported when metformin was combined with targeted or immune-checkpoint therapy of MM with anti-PD-1/anti-CTLA-4 [343,344]. Metformin suppresses MM cell growth and motility through modulation of miR expression [345]. Metformin reduces STAT3 activity in cancer cells and CSCs including glioblastoma and MM [346,347,348]. Metformin-mediated STAT3 suppression explains its inhibitory effect on miR-21 [349,350,351]. Metformin treatment in T2DM patients significantly reduces plasma levels of miR-21 and reduces miR-21 expression of CSCs [352,353].

### 6.3. Beta-Blocker

Beta-adrenoceptors (B_1–2_-AR) have emerged as novel targets to inhibit tumor growth and dissemination in cutaneous and uveal MM [354,355,356]. Beta-blocker use correlates with better overall survival in metastatic MM, protects patients from disease recurrence and improves the efficacy of immunotherapies in mice [354,355,356]. Catecholamine stimulation of B_1-2_-AR via activation of STAT3 upregulates miR-21 expression [357,358,359,360,361,362,363,364], whereas beta-blocker treatment attenuates miR-21 expression [364]. Beta-blocker-mediated reduction of miR-21 expression may also explain their anti-angiogenic effects in infantile hemangioma [365].

### 6.4. Anti-MiR-21

Direct targeting miR-21 has been suggested as a novel strategy for the treatment of cutaneous MM [33]. Anti-miR-21 mesyl phosphoramidate oligodeoxynucleotide specifically decreases miR-21 in melanoma B16 cells, induces apoptosis, reduces proliferation, and impedes migration of tumor cells [366]. Catalytic knockdown of miR-21 by artificial ribonuclease is another new option for tumors overexpressing miR-21 [367]. Adeno-associated viral vectors that preferentially express antisense miR against miR-21 has therapeutic efficacy in vivo in various cancer including glioblastoma [368]. Furthermore, miR-21 inhibitor and doxorubicin loaded nanometer (DLN) exert a favorable anti-cancer effect compared with single application of DLN or miR-21 inhibitor, respectively [369]. Of interest, miR-21 antisense oligonucleotide decreased IC50 and increased cisplatin sensitivity for A375 melanoma cells and A375/CDDP cells, which shows that miR-21 is a new target of MM treatment [370]. Furthermore, co-delivery of anti-miR-21 with doxorubicin prodrug by high-density lipoprotein-mimicking nanoparticles exerted a synergistic effect against drug resistance in cancer cells [371]. In accordance, co-delivery of 5-fluorouracil (5-FU) and miR-21 inhibitor oligonucleotide (miR-21i) with engineered exosomes to colorectal cancer cells (HCT-1165FR) significantly down-regulated miR-21, induced cell cycle arrest, reduced tumor proliferation, increased apoptosis and rescued PTEN and hMSH2 expressions, regulatory targets of miR-21 [372]. The combined delivery of miR-21i and 5-FU with engineered exosomes effectively reversed drug resistance and significantly enhanced the cytotoxicity in 5-FU-resistant colon cancer cells, compared with the single treatment with either miR-21i or 5-FU [372]. Recent studies support the combination of miR-21 inhibition and immune checkpoint blockade to target the MM microenvironment [38]. Thus, the application of exosomes loaded with anti-miR-21 may exert beneficial effects in the treatment of MM.

### 6.5. Interferons

Pegylated interferon-α (IFNα), as studied in the European Organisation for Research and Treatment of Cancer (EORTC) 18991 trial, in patients with stage III MM significantly reduced the risk of relapse (HR 0.87), however showed no impact on overall survival [373]. Treatment of dendritic cells (DCs) with IFNα-2b significantly upregulates surface expression of PD-L1 molecules and reduces the capacity to stimulate interferon-γ (IFN-γ) production in T cells compared to control DCs [374]. IFNα regulates PD-L1 expression through the STAT3 and p38 signaling pathways, since blocking of STAT3 and p38 activation with specific inhibitors prevents PD-L1 upregulation [374]. B16 mouse MM cells treated with IFNα (1000 IU/mL, for 6 h) exhibit a significant upregulation of miR-21 expression [69]. A miR microarray analysis confirmed that IFN-γ also upregulates miR-21 expression in A375 melanoma cells [375]. Thus, IFN treatment of metastatic MM has an undesirable impact on miR-21 expression.

### 6.6. High-Intensity Focused Ultrasound

High-intensity focused ultrasound (HIFU), a potential noninvasive treatment procedure for solid tumors, suppresses miR-21 expression, cell migration and metastasis in a murine MM model [376]. HIFU may disintegrate exosome membranes or disturb exosomal miR-21 traffic.

### 6.7. Iontophoretic Co-Delivery of STAT3 siRNA and Imatinib

Effective MM suppression has also been reported by iontophoretic co-delivery of STAT3 siRNA and imatinib using gold nanoparticles [377]. It is conceivable that this regimen might also attenuate miR-21 expression. Low expression of lncRNA MEG3 was associated with imatinib resistance and high miR-21 expression in chronic myeloid leukemia [378].

### 6.8. Curcumin

Curcumin, a natural compound derived from *Curcuma longa*, exerts anti-cancer properties, observed also in MM [379,380]. The pharmacological activity of curcumin is mediated by suppression of JAK2/STAT3 signaling, which induces miR-21 expression. In addition, curcumin inhibits NF-κB and directly binds to AP-1 at the *MIR21* promoter, thereby inhibiting AP-1-dependent expression of miR-21 [380,381,382,383].

### 6.9. Sulforaphane

Sulforaphane (SFN), an isothiocyanate found in cruciferous vegetables, when orally administered in broccoli sprout extract form, is well tolerated up to 200 μmol/day and dose-dependently increases SFN levels in plasma and skin of patients with atypical nevi and/or a prior history of MM [384]. In human MM cells, SFN causes cell cycle growth arrest and induces apoptosis [385,386,387]. Moreover, SFN is a potent histone deacetylase inhibitor, which via epigenetic mechanisms reduces miR-21 expression [388,389].

### 6.10. Epigallocatechin-3-Gallate

Epigallocatechin-3-gallate (EGCG), from *Camellia sinensis*, suppresses MM cell growth and metastasis [390,391,392,393,394]. Its anti-inflammatory and anti-proliferative activity is related to its suppressive effect on NF-κB and AP-1 [395,396], as a potential explanation for its suppressive effect on miR-21 expression [397,398].

### 6.11. Vitamin D

As outlined above, VD deficiency is related to poorer survival in metastatic MM [282,283]. Enhanced VDR signaling may reduce the expression of oncogenic miR-21 [54,94,95,307,308,309,310,311,312]. Nevertheless, VD supplementation for MM prevention and adjuvant therapy is still controversial [310,399,400,401,402], but has been recommended in a recent vitamin D symposium [403].

### 6.12. Exercise

A population-based study of 1.44 million Americans and Europeans suggested that leisure time physical activity is associated with a slightly higher risk of MM [404], though other studies have found an inverse relationship [405,406]. These studies are likely confounded by sun exposure. Several studies have shown that exercise has several anti-cancer effects [407]. In accordance, hormone therapy plus interval training significantly reduced tumor size and miR-21 levels in a murine breast tumor model [334]. Interestingly, 12 weeks of endurance training led to a significant decrease of miR-21 plasma levels in humans [335] (Table 5).

## 7. Conclusions and Perspectives

Scientific interest in oncogenic dysregulation of MM focuses mainly on DNA base mutations [408,409,410,411], primarily driven by UV-induced DNA damage [412]. In this review, translational and epidemiological evidence underlines that not only DNA base mutations, but also miR-21-mediated epigenetic alterations enhance NRAS, BRAF and downstream MEK-ERK and AKT-mTORC1 signaling in MM (Figure 2). Notably, miR-21 via targeting a variety of tumor suppressors activates proliferation pathways promoted by common MM gene mutations. There is accumulating evidence that miR-21 levels in melanocytes and MM cells are not only regulated by endogenous synthesis but also by exogenous transfer of miR-21-enriched exosomes secreted by various cells of the MM microenvironment as well as exosomes of the systemic circulation (Figure 4). Environmental and metabolic factors including UV, ionizing and electromagnetic radiation, electromagnetic irradiation, diet, smoking, air pollution and individual factors including birth weight, growth trajectories during infancy, obesity and hormonal factors may synergistically enhance the burden of miR-21 expression. Recent progress in exosome-dependent cell signaling opens new avenues in understanding the interaction of exosomal miR-21 trafficking to melanocytes as well as MM-derived exosome signaling with cells of the tumor environment. Various environmental impacts on melanomagenesis converge in upregulated exosomal miR-21 signaling [151], which finally modifies miR-21 homeostasis in cutaneous melanocytes (Figure 4). The missing correlation between miR-21 levels and NRAS and BRAF mutations in MM cells points to a mutation-independent pathway of miR-21-mediated signal transduction [39].

Therefore, therapeutic agents that attenuate miR-21 expression such as metformin, beta-blockers, vitamin D, curcumin, EGCG and SFN may have beneficial effects in MM prevention and treatment. The anti-proliferative effects of miR-21-antagonizing agents, its catalytic knockdown of miR-21 by artificial ribonuclease, and miR-21 expression lowering agents may synergistically improve MM therapy [365,366,367,368,369,370,371,413]. Delivery of exosomal anti-miR-21 may be a promising new option for MM treatment [367,368,369,370]. The fact that VD/VDR signaling attenuates miR-21 expression [305,306,307] suggests that VD deficient individuals should be given oral VD supplementation, and questions extensive use of sunscreens for MM prevention. Obviously, there should be an appropriate balance between UV-induced exosomal miR-21 expression and UV-stimulated vitamin D/VDR-mediated attenuation of miR-21 expression.

Future studies should characterize the epigenetic impact of Western lifestyle factors on aberrant exosomal miR-21 signaling in MM (Figure 4). A deeper understanding of the various cellular origins of miR-21-enriched exosomes on melanomagenesis and MM progression will hopefully open new avenues for the prevention and successful management of MM. Delivery of exosomal anti-miR-21 or interruption of MM exosome traffic, as well as attenuation of cellular miR-21 expression, may be promising future strategies for MM therapy.

## Figures and Tables

**Table 1:** MiR-21 expression in melanoma (MM) and related pathological effects.

Associations of miR-21 with Melanoma Pathology	References
MiR-21 expression increases from benign nevi to MM and metastatic MM	[31,33]
MiR-21 expression correlates with mitotic activity in MM	[34]
MiR-21 levels correlate with MM cellularity	[36]
MiR-21 promotes proliferation, migration, and inhibits apoptosis of MM cells	[31,32]
MiR-21 expression correlates with Breslow thickness and advanced clinical stage	[31,33]
MiR-21 expression correlates with positive sentinel lymph node biopsy	[34]
MiR-21 promotes MM invasion and metastasis	[37]
MiR-21 expression correlates with shorter 5-year disease-free or overall survival	[33]
MiR-21 inhibits PD-L1 expression of MM-associated macrophages	[38]
Antisense-mediated miR-21 inhibition suppresses growth, increases apoptosis and enhances chemo- or radiosensitivity of human MM cells	[33]

PD-L1: programmed death ligand 1.

**Table 2:** MiR-21 target genes and their involvement in melanoma.

Target Genes	Proteins	Functions	References
*TIPE2*	Tumor necrosis factor-α-induced protein 8 (TNFAIP8)-like 2	Inhibition of RAS	[43,44,45]
*SPRY1*	Sprouty RTK signaling antagonist 1	Inhibition of RAS and RAF	[40,42]
*SPRY2*	Sprouty RTK signaling antagonist 2	Inhibition of RAS and RAF	[42,49]
*PTEN*	Phosphatase and tensin homolog	Inhibition of PI3K and downstream AKT-mTORC1 signaling	[40,49]
*PDCD4*	Programmed cell death 4	Inhibition of translation initiation	[40,49]
*FBXO11*	F-box only protein 11	Tumor suppression promoting apoptosis, exhibiting decreased expression in higher Clark level MM	[50]
*SOX5*	SRY-box 5	Suppression of MITF	[51,52]
*CDKN2C*	Cyclin-dependent kinase inhibitor 2C	Inhibition of G_1_/S transition, proliferation	[32,49]
*MSH2*	DNA mismatch repair protein 2	DNA repair, prevention of microsatellite instability	[49,53]
*CYP27B1*	25-hydroxyvitamin D_3_-1-α-hydroxylase	Conversion of 25(OH) vitamin D to active 1,25(OH)_2_ vitamin D_3_	[54]
*RECK*	Reversion-inducing cysteine-rich protein with Kazal motifs	Extracellular matrix integrity and regulation of angiogenesis	[49,55]
*TIMP1*	Tissue inhibitor of metalloproteinase 1	Inhibition of MMP1-mediated matrix degradation	[49,56]
*TIMP3*	Tissue inhibitor of metalloproteinase 3	Inhibition of MMP3-mediated matrix degradation	[49,57]
*CDH1*	E-cadherin	Cell-cell adhesion	[58]
*IL12A*	p35 subunit of interleukin 12	Anti-tumor activities via NK- and cytotoxic T cell activation	[59]
*AKT1S1*	AKT1 substrate 1, proline-rich	Negative regulator of mTORC1	[60]

RTK: receptor tyrosine kinase; PI3K: phosphytidylinositol-3 kinase; mTORC1: mechanistic target of rapamaycin complex 1; MITF: microphthalmia-associated transcription factor; MMP: matrix metallo- proteinase; NK cell: natural killer T cell.

**Table 3:** Regulators of miR-21 expression.

Regulatory Agent	Transcriptional Regulator	MiR-21 Expression	References
AP1	Activator protein 1 (Fos, Jun)	Upregulation	[25,92]
STAT3	Signal transducer and activator of transcription 3	Upregulation	[25,57,69,70,71]
p65	Nuclear factor kappa-B, subunit 3	Upregulation	[25]
AR	Androgen receptor	Upregulation	[25,93]
PU.1	ETS-domain transcription factor PU.1	Upregulation	[25]
C/EBPα	CCATT/enhancer binding protein α	Upregulation	[25]
TGFβ1	Transforming growth factor β1	Upregulation	[25]
NFIB	Nuclear factor IB	Downregulation	[25]
VDR	Vitamin D receptor	Downregulation	[94,95]
XIST	X inactivation-specific transcript (lncRNA)	Downregulation	[79]
MEG3	Maternally expressed gene 3 (lncRNA)	Downregulation	[58]
GAS5	Growth arrest specific transcript 5 (lncRNA)	Downregulation	[87,88,89]

**Table 4:** Lifestyle factors upregulating miR-21 expression.

Lifestyle Factor	Biological Responses	References
Ultraviolet radiation	Keratinocyte-derived release of miR-21-enriched exosomes; increased miR-21 expression of melanocytes	[163,164,165,166,167]
Cosmic irradiation	Increase of exosomal miR-21	[177,178,179,180,181]
Electromagnetic radiation	Increased expression of miR-21	[183]
Obese adipose tissue	Adipocyte secretome with increased release of miR-21-enriched exosomes	[232,233,234,235,236]
High-fat diet-induced obesity	Increase of circulatory and adipocyte miR-21	[230,231]
High glucose intake	Increase of circulatory miR-21	[60]
High fructose intake	Increase of circulatory miR-21	[256]
Alcohol consumption	Increase of circulatory miR-21	[276,277]
Milk consumption	Increase of circulatory exosomal miR-21	[210,211,212,213]
Vitamin D deficiency	Increased expression of miR-21	[95]
Smoking	Increased expression of exosomal miR-21 in airway epithelial cells	[287,288,289]
Air pollution (Diesel)	Increased expression of exosomal miR-21 in airway epithelial cells	[290]
Sedentary lifestyle	Increase of circulatory miR-21	[334,335]
Aging	Increase of circulatory miR-21	[324,325]
Chronic inflammation	Increase of circulatory miR-21	[331,332,333]

**Table 5:** Therapeutic interventions attenuating miR-21 expression.

Therapeutic Factors	Potential Benefits for Melanoma Prevention and Therapy	References
Anti-miR-21	Direct suppression of miR-21 signaling in melanocytes, activation of skin-resident CD8+ memory T-cells; reduction of miR-21 in tumor-associated macrophages associated with improved cytotoxic T-cell responses	[33,366,367,368,369,370,371]
BRAF inhibition	Attenuation of miR-21 expression	[336]
Sunscreen	Reduction of keratinocyte-derived exosomal miR-21	[163,164,165,166,167]
Restriction of electro-magnetic radiation	Limitation of smart phone radiation on miR-21 expression	[189,190]
Control of birth weight and body weight	Balanced expression of miR-21 during fetal and postnatal life	[203,204]
Reduction of glycemic load and fat intake	Reduction of circulating and adipocyte-derived miR-21	[60,230,231,256]
Cessation of smoking	Reduction of airway epithelial cell-derived exosomal miR-21	[287,288,289]
Restriction of alcohol intake	Reduction of miR-21 expression	[276,277]
Metformin	Reduction of STAT3 activation and miR-21 expression	[349,350,352,353]
Beta-blocker	Suppression of STAT3 activation and miR-21 expression	[364]
Curcumin	Suppression of STAT3 activation and inactivation of AP-1 resulting in reduced expression of miR-21	[380,381,382,383]
EGCG	Reduction of miR-21 expression	[397,398]
Sulforaphane	Reduction of miR-21 expression	[388,389]
Vitamin D	Reduction of miR-21 expression	[54,95,307]
Exercise	Reduction of miR-21 expression	[334,335]
HIFU	Reduction of miR-21 in metastatic melanoma tissue	[376]

## Abbreviations

AKT V-AKT murine thymoma viral oncogene homolog, AP-1 activator protein 1, AR androgen receptor, BAX BCL2-associated X protein, BCL2 B-cell CLL/lymphoma 2, BRAF V-RAF murine sarcoma viral oncogene homolog B, BTG2 B-cell translocation gene 2, BW birth weight, CCA cancer-associated adipocyte, CDKN2C cyclin-dependent kinase inhibitor 2C, CYP27B1 25-hydroxyvitamin D_3_-1-hydroxylase, DICER1 ribonuclease III, DLN doxorubicin loaded nanometer, EGCG epigallocatechin-3-gallate, EGFR epidermal growth factor receptor, EMT epithelial to mesenchymal transition, ERK extracellular signal-related kinase, FASLG Fas ligand, FBXO11 F-box only protein 11, FFA free fatty acid, FM frequency modulation, GAS5 long non-coding RNA growth arrest-specific transcript 5, GH growth hormone, GHR growth hormone receptor, GPCR G protein-coupled receptor, HFD high-fat diet, HIF-1α hypoxia-inducible factor 1 α-subunit, HIFU high intensity focused ultrasound, HMG-CoA 3-hydroxy-3-methylglataryl-CoA, hTERT human telomerase reverse transcriptase, ICI immune-checkpoint inhibition, JAK2 Janus kinase 2, KIT V-KIT Hardy-Zuckerman 4 feline sarcoma viral oncogene homolog, LET linear energy transfer, LNA locked nucleic acid, MEG3 long non-coding RNA maternally expressed gene 3, MEK1 mitogen-activated protein kinase kinase 1, MiR-21 micro-ribonucleic acid 21, MITF microphthalmia-associated transcription factor, MM malignant melanoma, MMR DNA mismatch repair, MSH2 MutS homolog 2, mTORC1 mechanistic target of rapamycin complex 1, NEDD9 neural precursor cell expressed developmentally downregulated 9, NF-κB nuclear factor-κB, NKG2D killer cell lectin-like, subfamily K member 1, NRAS NRAS protooncogene GTPase, PDCD4 programmed cell death 4, PD-L1 programmed death ligand 1, PI3K phosphatidylinositol-3 kinase, PRAS40 proline-rich AKT substrate 40-KD, PTEN phosphatase and tensin homolog, RECK reversion-inducing cysteine-rich protein with Kazal motifs, SFN sulforaphane, siRNA small interfering RNA, SLNB sentinel lymph node biopsy, SOX5 SRY-box 5, SRC V-SRC avian sarcoma (Schmidt-Ruppin A-2) viral oncogene, SPRY Sprouty RTK signaling antagonist, STAT3 signal transducer and activator of transcription 3, TAZ transcriptional coactivator with PDZ-binding motif, TCR T-cell receptor, T1DM type 1 diabetes mellitus, T2DM type 2 diabetes mellitus, TIM tumor infiltrating myeloid cell, TIMP3 tissue inhibitor of metalloproteinase 3, TIPE2 tumor necrosis factor-α-induced protein 8-like 2, TRM tissue-resident CD8+ memory T cells, UV ultraviolet, UV-A ultraviolet A radiation, UV-B ultraviolet B radiation, VD vitamin D, VDR vitamin D receptor, VEGF vascular endothelial growth factor, XIST X-inactive-specific transcript, YAP yes-associated protein.

## References

[1] Rastrelli M., Tropea S., Rossi C.R., Alaibac M. (2014). Melanoma: Epidemiology, risk factors, pathogenesis, diagnosis and classification. In Vivo.

[2] Hallberg O., Johansson O. (2004). Malignant melanoma of the skin—Not a sunshine story!. Med. Sci. Monit..

[3] Whiteman D.C., Green A.C., Olsen C.M. (2016). The growing burden of invasive melanoma: Projections of incidence rates and numbers of new cases in six susceptible populations through 2031. J. Investig. Dermatol..

[4] Garbe C., Keim U., Eigentler T.K., Amaral T., Katalinic A., Holleczek B., Martus P., Leiter U. (2019). Time trends in incidence and mortality of cutaneous melanoma in Germany. J. Eur. Acad. Dermatol. Venereol..

[5] Garraway L.A., Widlund H.R., Rubin M.A., Getz G., Berger A.J., Ramaswamy S., Beroukhim R., Milner D.A., Granter S.R., Du J. (2005). Integrative genomic analyses identify MITF as a lineage survival oncogene amplified in malignant melanoma. Nature.

[6] Berger M.F., Garraway L.A. (2009). Applications of genomics in melanoma oncogene discovery. Hematol. Oncol. Clin. N. Am..

[7] Bastian B.C. (2014). The molecular pathology of melanoma: An integrated taxonomy of melanocytic neoplasia. Annu. Rev. Pathol..

[8] Kunz M. (2014). Oncogenes in melanoma: An update. Eur. J. Cell Biol..

[9] Leonardi G.C., Falzone L., Salemi R., Zanghì A., Spandidos D.A., Mccubrey J.A., Candido S., Libra M. (2018). Cutaneous melanoma: From pathogenesis to therapy (Review). Int. J. Oncol..

[10] Lorentzen H.F. (2019). Targeted therapy for malignant melanoma. Curr. Opin. Pharmacol..

[11] Reu F.J., Bae S.I., Cherkassky L., Leaman D.W., Lindner D., Beaulieu N., MacLeod A.R., Borden E.C. (2006). Overcoming resistance to interferon-induced apoptosis of renal carcinoma and melanoma cells by DNA demethylation. J. Clin. Oncol..

[12] De Vries I.J., Castelli C., Huygens C., Jacobs J.F., Stockis J., Schuler-Thurner B., Adema G.J., Punt C.J., Rivoltini L., Schuler G. (2011). Frequency of circulating Tregs with demethylated FOXP3 intron 1 in melanoma patients receiving tumor vaccines and potentially Treg-depleting agents. Clin. Cancer Res..

[13] Micevic G., Theodosakis N., Bosenberg M. (2017). Aberrant DNA methylation in melanoma: Biomarker and therapeutic opportunities. Clin. Epigenet..

[14] De Unamuno Bustos B., Murria Estal R., Pérez Simó G., Simarro Farinos J., Pujol Marco C., Navarro Mira M., Alegre de Miquel V., Ballester Sánchez R., Sabater Marco V., Llavador Ros M. (2018). Aberrant DNA methylation is associated with aggressive clinicopathological features and poor survival in cutaneous melanoma. Br. J. Dermatol..

[15] Bonazzi V.F., Stark M.S., Hayward N.K. (2012). MicroRNA regulation of melanoma progression. Melanoma Res..

[16] Völler D., Ott C., Bosserhoff A. (2013). MicroRNAs in malignant melanoma. Clin. Biochem..

[17] Luo C., Weber C.E., Osen W., Bosserhoff A.K., Eichmüller S.B. (2014). The role of microRNAs in melanoma. Eur. J. Cell Biol..

[18] Sun V., Zhou W.B., Majid S., Kashani-Sabet M., Dar A.A. (2014). MicroRNA-mediated regulation of melanoma. Br. J. Dermatol.

[19] Sarkar D., Leung E.Y., Baguley B.C., Finlay G.J., Askarian-Amiri M.E. (2016). Epigenetic regulation in human melanoma: Past and future. Epigenetics.

[20] Mirzaei H., Gholamin S., Shahidsales S., Sahebkar A., Jaafari M.R., Mirzaei H.R., Hassanian S.M., Avan A. (2016). MicroRNAs as potential diagnostic and prognostic biomarkers in melanoma. Eur. J. Cancer.

[21] Fattore L., Costantini S., Malpicci D., Ruggiero C.F., Ascierto P.A., Croce C.M., Mancini R., Ciliberto G. (2017). MicroRNAs in melanoma development and resistance to target therapy. Oncotarget.

[22] Sánchez-Sendra B., Martinez-Ciarpaglini C., González-Muñoz J.F., Murgui A., Terrádez L., Monteagudo C. (2018). Downregulation of intratumoral expression of miR-205, miR-200c and miR-125b in primary human cutaneous melanomas predicts shorter survival. Sci. Rep..

[23] Varrone F., Caputo E. (2020). The miRNAs role in melanoma and in its resistance to therapy. Int. J. Mol. Sci..

[24] Feng Y.H., Tsao C.J. (2016). Emerging role of microRNA-21 in cancer. Biomed. Rep..

[25] Pan X., Wang Z.X., Wang R. (2010). MicroRNA-21: A novel therapeutic target in human cancer. Cancer Biol. Ther..

[26] Latchana N., Del Campo S.E., Grignol V.P., Clark J.R., Albert S.P., Zhang J., Wei L., Aldrink J.H., Nicol K.K., Ranalli M.A. (2017). Classification of indeterminate melanocytic lesions by microRNA profiling. Ann. Surg. Oncol..

[27] Wandler A., Riber-Hansen R., Hager H., Hamilton-Dutoit S.J., Schmidt H., Nielsen B.S., Stougaard M., Steiniche T. (2017). Quantification of microRNA-21 and microRNA-125b in melanoma tissue. Melanoma Res..

[28] Ferracin M., Lupini L., Salamon I., Saccenti E., Zanzi M.V., Rocchi A., Da Ros L., Zagatti B., Musa G., Bassi C. (2015). Absolute quantification of cell-free microRNAs in cancer patients. Oncotarget.

[29] Carpi S., Polini B., Fogli S., Podestà A., Ylösmäki E., Cerullo V., Romanini A., Nieri P. (2020). Circulating microRNAs as biomarkers for early diagnosis of cutaneous melanoma. Expert Rev. Mol. Diagn..

[30] Neagu M., Constantin C., Cretoiu S.M., Zurac S. (2020). miRNAs in the diagnosis and prognosis of skin cancer. Front. Cell Dev. Biol..

[31] Satzger I., Mattern A., Kuettler U., Weinspach D., Niebuhr M., Kapp A., Gutzmer R. (2012). microRNA-21 is upregulated in malignant melanoma and influences apoptosis of melanocytic cells. Exp. Dermatol..

[32] Yang Z., Liao B., Xiang X., Ke S. (2020). MiR-21-5p promotes cell proliferation and G1/S transition in melanoma by targeting CDKN2C. FEBS Open Bio.

[33] Jiang L., Lv X., Li J., Li J., Li X., Li W., Li Y. (2012). The status of microRNA-21 expression and its clinical significance in human cutaneous malignant melanoma. Acta Histochem..

[34] Grignol V., Fairchild E.T., Zimmerer J.M., Lesinski G.B., Walker M.J., Magro C.M., Kacher J.E., Karpa V.I., Clark J., Nuovo G. (2011). miR-21 and miR-155 are associated with mitotic activity and lesion depth of borderline melanocytic lesions. Br. J. Cancer.

[35] Yang C., Wie W. (2011). The miRNA expression profile of the uveal melanoma. Sci. China Life Sci..

[36] Torres R., Lang U.E., Hejna M., Shelton S.J., Joseph N.M., Shain A.H., Yeh I., Wei M.L., Oldham M.C., Batian B.C. (2020). MicroRNA ratios distinguish melanomas from nevi. J. Investig. Dermatol..

[37] Mo H., Guan J., Yuan Z.C., Lin X., Wu Z.J., Liu B., He J.L. (2019). Expression and predictive value of miR-489 and miR-21 in melanoma metastasis. World J. Clin. Cases.

[38] Xi J., Huang Q., Wang L., Ma X., Deng Q., Kumar M., Zhou Z., Li L., Zeng Z., Young K.H. (2018). miR-21 depletion in macrophages promotes tumoricidal polarization and enhances PD-1 immunotherapy. Oncogene.

[39] Saldanha G., Potter L., Lee Y.S., Watson S., Shendge P., Pringle J.H. (2016). MicroRNA-21 expression and its pathogenetic significance in cutaneous melanoma. Melanoma Res..

[40] Mao X.H., Chen M., Wang Y., Cui P.G., Liu S.B., Xu Z.Y. (2017). MicroRNA-21 regulates the ERK/NF-κB signaling pathway to affect the proliferation, migration, and apoptosis of human melanoma A375 cells by targeting SPRY1, PDCD4, and PTEN. Mol. Carcinog..

[41] Wang J.H., Zheng W.W., Cheng S.T., Liu B.X., Liu F.R., Song J.Q. (2015). Correlation between microRNA-21 and sprouty homolog 2 gene expression in multiple myeloma. Mol. Med. Rep..

[42] Liu Z., Liu X., Cao W., Hua Z.C. (2015). Tumor-specifically hypoxia-induced therapy of SPRY1/2 displayed differential therapeutic efficacy for melanoma. Am. J. Cancer Res..

[43] Ruan Q., Wang P., Wang T., Qi J., Wei M., Wang S., Fan T., Johnson D., Wan X., Shi W. (2014). MicroRNA-21 regulates T-cell apoptosis by directly targeting the tumor suppressor gene Tipe2. Cell Death Dis..

[44] Li Z., Jia W., Niu J., Zhang L. (2018). Understanding the roles of negative immune regulator TIPE2 in different diseases and tumourigenesis. Histol. Histopathol..

[45] Gus-Brautbar Y., Johnson D., Zhang L., Sun H., Wang P., Zhang S., Zhang L., Chen Y.H. (2012). The anti-inflammatory TIPE2 is an inhibitor of the oncogenic Ras. Mol. Cell..

[46] Lito P., Pratilas C.A., Joseph E.W., Tadi M., Halilovic E., Zubrowski M., Huang A., Wong W.L., Callahan M.K., Merghoub T. (2012). Relief of profound feedback inhibition of mitogenic signaling by RAF inhibitors attenuates their activity in BRAFV600E melanomas. Cancer Cell..

[47] Hatley M.E., Patrick D.M., Garcia M.R., Richardson J.A., Bassel-Duby R., van Rooij E., Olson E.N. (2010). Modulation of K-Ras-dependent lung tumorigenesis by microRNA-21. Cancer Cell.

[48] Boiko A.D., Porteous S., Razorenova O.V., Krivokrysenko V.I., Williams B.R., Gudkov A.V. (2006). A systematic search for downstream mediators of tumor suppressor function of p53 reveals a major role of BTG2 in suppression of Ras-induced transformation. Genes Dev..

[49] Buscaglia L.E., Li Y. (2011). Apoptosis and the target genes of microRNA-21. Chin. J. Cancer.

[50] Yang C.H., Pfeffer S.R., Sims M., Yue J., Wang Y., Linga V.G., Paulus E., Davidoff A.M., Pfeffer L.M. (2015). The oncogenic microRNA-21 inhibits the tumor suppressive activity of FBXO11 to promote tumorigenesis. J. Biol. Chem..

[51] Wang P., Zhao Y., Fan R., Chen T., Dong C. (2016). MicroRNA-21a-5p functions on the regulation of melanogenesis by targeting Sox5 in mouse skin melanocytes. Int. J. Mol. Sci..

[52] Kordaß T., Weber C.E., Oswald M., Ast V., Bernhardt M., Novak D., Utikal J., Eichmüller S.B., König R. (2016). SOX5 is involved in balanced MITF regulation in human melanoma cells. BMC Med. Genomics..

[53] Bhandari A., Gordon W., Dizon D., Hopkin A.S., Gordon E., Yu Z., Andersen B. (2013). The Grainyhead transcription factor Grhl3/Get1 suppresses miR-21 expression and tumorigenesis in skin: Modulation of the miR-21 target MSH2 by RNA-binding protein DND1. Oncogene.

[54] Liu P.T., Wheelwright M., Teles R., Komisopoulou E., Edfeldt K., Ferguson B., Mehta M.D., Vazirnia A., Rea T.H., Sarno E.N. (2012). MicroRNA-21 targets the vitamin D-dependent antimicrobial pathway in leprosy. Nat. Med..

[55] Reis S.T., Pontes-Junior J., Antunes A.A., Dall′Oglio M.F., Dip N., Passerotti C.C., Rossini G.A., Morais D.R., Nesrallah A.J., Piantino C. (2012). miR-21 may acts as an oncomir by targeting RECK, a matrix metalloproteinase regulator, in prostate cancer. BMC Urol..

[56] Martin del Campo S.E., Latchana N., Levine K.M., Grignol V.P., Fairchild E.T., Jaime-Ramirez A.C., Dao T.V., Karpa V.I., Carson M., Ganju A. (2015). MiR-21 enhances melanoma invasiveness via inhibition of tissue inhibitor of metalloproteinases 3 expression: In vivo effects of miR-21 inhibitor. PLoS ONE.

[57] Gutsaeva D.R., Thounaojam M., Rajpurohit S., Powell F.L., Martin P.M., Goei S., Duncan M., Bartoli M. (2017). STAT3-mediated activation of miR-21 is involved in down-regulation of TIMP3 and neovascularization in the ischemic retina. Oncotarget.

[58] Wu L., Zhu L., Li Y., Zheng Z., Lin X., Yang C. (2020). LncRNA MEG3 promotes melanoma growth, metastasis and formation through modulating miR-21/E-cadherin axis. Cancer Cell Int..

[59] Lu T.X., Munitz A., Rothenberg M.E. (2009). MicroRNA-21 is up-regulated in allergic airway inflammation and regulates IL-12p35 expression. J. Immunol..

[60] Dey N., Das F., Mariappan M.M., Mandal C.C., Ghosh-Choudhury N., Kasinath B.S., Choudhury G.G. (2011). MicroRNA-21 orchestrates high glucose-induced signals to TOR complex 1, resulting in renal cell pathology in diabetes. J. Biol. Chem..

[61] Iliopoulos D., Jaeger S.A., Hirsch H.A., Bulyk M.L., Struhl K. (2010). STAT3 activation of miR-21 and miR-181b-1 via PTEN and CYLD are part of the epigenetic switch linking inflammation to cancer. Mol. Cell..

[62] Logotheti S., Pützer B.M. (2019). STAT3 and STAT5 targeting for simultaneous management of melanoma and autoimmune diseases. Cancers.

[63] Huynh J., Etemadi N., Hollande F., Ernst M., Buchert M. (2017). The JAK/STAT3 axis: A comprehensive drug target for solid malignancies. Semin. Cancer Biol..

[64] Sun X., Cao N., Mu L., Cao W. (2019). Stress induced phosphoprotein 1 promotes tumor growth and metastasis of melanoma via modulating JAK2/STAT3 pathway. Biomed. Pharmacother..

[65] Kulesza D.W., Przanowski P., Kaminska B. (2019). Knockdown of STAT3 targets a subpopulation of invasive melanoma stem-like cells. Cell. Biol. Int..

[66] Wang X., Qu H., Dong Y., Wang G., Zhen Y., Zhang L. (2018). Targeting signal-transducer-and-activator-of-transcription 3 sensitizes human cutaneous melanoma cells to BRAF inhibitor. Cancer Biomark..

[67] Pan J., Ruan W., Qin M., Long Y., Wan T., Yu K., Zhai Y., Wu C., Xu Y. (2018). Intradermal delivery of STAT3 siRNA to treat melanoma via dissolving microneedles. Sci. Rep..

[68] Delyon J., Chevret S., Jouary T., Dalac S., Dalle S., Guillot B., Arnault J.P., Avril M.F., Bedane C., Bens G. (2018). STAT3 mediates nilotinib response in KIT-altered melanoma: A phase II multicenter trial of the French Skin Cancer Network. J. Investig. Dermatol..

[69] Yang C.H., Yue J., Fan M., Pfeffer L.M. (2010). IFN induces miR-21 through a signal transducer and activator of transcription 3-dependent pathway as a suppressive negative feedback on IFN-induced apoptosis. Cancer Res..

[70] Tscherner A., Brown A.C., Stalker L., Kao J., Dufort I., Sirard M.A., LaMarre J. (2018). STAT3 signaling stimulates miR-21 expression in bovine cumulus cells during in vitro oocyte maturation. Sci. Rep..

[71] Yang C.H., Yue J., Pfeffer S.R., Handorf C.R., Pfeffer L.M. (2011). MicroRNA miR-21 regulates the metastatic behavior of B16 melanoma cells. J. Biol. Chem..

[72] Koo J.H., Plouffe S.W., Meng Z., Lee D.H., Yang D., Lim D.S., Wang C.Y., Guan K. (2020). Induction of AP-1 by YAP/TAZ contributes to cell proliferation and organ growth. Genes Dev..

[73] Nallet-Staub F., Marsaud V., Li L., Gilbert C., Dodier S., Bataille V., Sudol M., Herlyn M., Mauviel A. (2014). Pro-invasive activity of the Hippo pathway effectors YAP and TAZ in cutaneous melanoma. J. Investig. Dermatol..

[74] Zhang X., Tang J.Z., Vergara I.A., Zhang Y., Szeto P., Yang L., Mintoff C., Colebatch A., McIntosh L., Mitchell K.A. (2019). Somatic hypermutation of the YAP oncogene in a human cutaneous melanoma. Mol. Cancer Res..

[75] Li H., Li Q., Dang K., Ma S., Cotton J.L., Yang S., Zhu L.J., Deng A.C., Ip Y.T., Johnson R.L. (2019). YAP/TAZ activation drives uveal melanoma initiation and progression. Cell Rep..

[76] Menzel M., Meckbach D., Weide B., Toussaint N.C., Schilbach K., Noor S., Eigentler T., Ikenberg K., Busch C., Quintanilla-Martinez L. (2014). In melanoma, Hippo signaling is affected by copy number alterations and YAP1 overexpression impairs patient survival. Pigment Cell Melanoma Res..

[77] Yu X., Zheng H., Tse G., Chan M.T., Wu W.K. (2018). Long non-coding RNAs in melanoma. Cell Prolif..

[78] Yang Z., Jiang X., Jiang X., Zhao H. (2018). X-inactive-specific transcript: A long noncoding RNA with complex roles in human cancers. Gene.

[79] Zhang R., Xia T. (2017). Long non-coding RNA XIST regulates PDCD4 expression by interacting with miR-21-5p and inhibits osteosarcoma cell growth and metastasis. Int. J. Oncol..

[80] Pan B.M., Lin X., Zhang L., Hong W., Zhang Y. (2019). Long noncoding RNA X-inactive specific transcript promotes malignant melanoma progression and oxaliplatin resistance. Melanoma Res..

[81] Yao Y., Ma J., Xue Y., Wang P., Li Z., Liu J., Chen L., Xi Z., Teng H., Wang Z. (2015). Knockdown of long non-coding RNA XIST exerts tumor-suppressive functions in human glioblastoma stem cells by up-regulating miR-152. Cancer Lett..

[82] Cheng Z., Luo C., Guo Z. (2020). LncRNA-XIST/microRNA-126 sponge mediates cell proliferation and glucose metabolism through the IRS1/PI3K/Akt pathway in glioma. J. Cell Biochem..

[83] Li J., Bian E.B., He X.J., Ma C.C., Zong G., Wang H.L., Zhao B. (2016). Epigenetic repression of long non-coding RNA MEG3 mediated by DNMT1 represses the p53 pathway in gliomas. Int. J. Oncol..

[84] Chen L., Yang H., Xiao Y., Tang X., Li Y., Han Q., Fu J., Yang Y., Zhu Y. (2016). LncRNA GAS5 is a critical regulator of metastasis phenotype of melanoma cells and inhibits tumor growth in vivo. Onco Targets Ther..

[85] Bian D., Shi W., Shao Y., Li P., Song G. (2017). Long non-coding RNA GAS5 inhibits tumorigenesis via miR-137 in melanoma. Am. J. Transl. Res..

[86] Chen L., Yang H., Xiao Y., Tang X., Li Y., Han Q., Fu J., Yang Y., Zhu Y. (2016). Lentiviral-mediated overexpression of long non-coding RNA GAS5 reduces invasion by mediating MMP2 expression and activity in human melanoma cells. Int. J. Oncol..

[87] Tao H., Zhang J.G., Qin R.H., Dai C., Shi P., Yang J.J., Deng Z.Y., Shi K.H. (2017). LncRNA GAS5 controls cardiac fibroblast activation and fibrosis by targeting miR-21 via PTEN/MMP-2 signaling pathway. Toxicology.

[88] Hu L., Ye H., Huang G., Luo F., Liu Y., Liu Y., Yang X., Shen J., Liu Q., Zhang J. (2016). Long noncoding RNA GAS5 suppresses the migration and invasion of hepatocellular carcinoma cells via miR-21. Tumour Biol..

[89] Zhang Z., Zhu Z., Watabe K., Zhang X., Bai C., Xu M., Wu F., Mo Y.Y. (2013). Negative regulation of lncRNA GAS5 by miR-21. Cell Death Differ..

[90] Fan Q., Yang L., Zhang X., Peng X., Wei S., Su D., Zhai Z., Hua X., Li H. (2018). The emerging role of exosome-derived non-coding RNAs in cancer biology. Cancer Lett..

[91] Wang M., Zhou L., Yu F., Zhang Y., Li P., Wang K. (2019). The functional roles of exosomal long non-coding RNAs in cancer. Cell Mol. Life Sci..

[92] Watanabe K., Narumi T., Watanabe T., Otaki Y., Takahashi T., Aono T., Goto J., Toshima T., Sugai T., Wanezaki M. (2020). The association between microRNA-21 and hypertension-induced cardiac remodeling. PLoS ONE.

[93] Ribas J., Ni X., Haffner M., Wentzel E.A., Salmasi A.H., Chowdhury W.H., Kudrolli T.A., Yegnasubramanian S., Luo J., Rodriguez R. (2009). miR-21: An androgen receptor-regulated microRNA that promotes hormone-dependent and hormone-independent prostate cancer growth. Cancer Res..

[94] Zhou Z., Li X., Jiang G., Wang J., Qian Y. (2019). Vitamin D down-regulates microRNA-21 expression to promote human placental trophoblast cell migration and invasion in vitro. Nan Fang Yi Ke Da Xue Xue Bao.

[95] Sheane B.J., Smyth P., Scott K., Aziz R., Buckley M., Lodge E., Kiely N., Kingston M., McGovern E., Healy M. (2015). An association between microRNA-21 expression and vitamin D deficiency in coronary artery disease. Microrna.

[96] Liu Y., Wang H., Wang J. (2018). Exosomes as a novel pathway for regulating development and diseases of the skin. Biomed. Rep..

[97] Wang W.M., Wu C., Jin H.Z. (2019). Exosomes in chronic inflammatory skin diseases and skin tumors. Exp. Dermatol..

[98] Xiao D., Ohlendorf J., Chen Y., Taylor D.D., Rai S.N., Waigel S., Zacharias W., Hao H., McMasters K.M. (2012). Identifying mRNA, microRNA and protein profiles of melanoma exosomes. PLoS ONE.

[99] Gowda R., Robertson B.M., Iyer S., Barry J., Dinavahi S.S., Robertson G.P. (2020). The role of exosomes in metastasis and progression of melanoma. Cancer Treat. Rev..

[100] Tian T., Zhu Y.L., Zhou Y.Y., Liang G.F., Wang Y.Y., Hu F.H., Xiao Z.D. (2014). Exosome uptake through clathrin-mediated endocytosis and macropinocytosis and mediating miR-21 delivery. J. Biol. Chem..

[101] Mannavola F., D′Oronzo S., Cives M., Stucci L.S., Ranieri G., Silvestris F., Tucci M. (2019). Extracellular vesicles and epigenetic modifications are hallmarks of melanoma progression. Int. J. Mol. Sci..

[102] Hood J.L. (2019). Natural melanoma-derived extracellular vesicles. Semin. Cancer Biol..

[103] Harmati M., Gyukity-Sebestyen E., Dobra G., Janovak L., Dekany I., Saydam O., Hunyadi-Gulyas E., Nagy I., Farkas A., Pankotai T. (2019). Small extracellular vesicles convey the stress-induced adaptive responses of melanoma cells. Sci. Rep..

[104] Hu T., Hu J. (2019). Melanoma-derived exosomes induce reprogramming fibroblasts into cancer-associated fibroblasts via Gm26809 delivery. Cell Cycle.

[105] Boussadia Z., Lamberti J., Mattei F., Pizzi E., Puglisi R., Zanetti C., Pasquini L., Fratini F., Fantozzi L., Felicetti F. (2018). Acidic microenvironment plays a key role in human melanoma progression through a sustained exosome mediated transfer of clinically relevant metastatic molecules. J. Exp. Clin. Cancer Res..

[106] Peinado H., Alečković M., Lavotshkin S., Matei I., Costa-Silva B., Moreno-Bueno G., Hergueta-Redondo M., Williams C., García-Santos G., Ghajar C. (2012). Melanoma exosomes educate bone marrow progenitor cells toward a pro-metastatic phenotype through MET. Nat. Med..

[107] Zhou X., Yan T., Huang C., Xu Z., Wang L., Jiang E., Wang H., Chen Y., Liu K., Shao Z. (2018). Melanoma cell-secreted exosomal miR-155-5p induce proangiogenic switch of cancer-associated fibroblasts via SOCS1/JAK2/STAT3 signaling pathway. J. Exp. Clin. Cancer Res..

[108] Abels E.R., Maas S.L.N., Nieland L., Wie Z., Cheah P.S., Tai E., Kolsteeg C.J., Dusoswa S.A., Ting D.T., Hickman S. (2019). Glioblastoma-associated microglia reprogramming is mediated by functional transfer of extracellular miR-21. Cell Rep..

[109] Gajos-Michniewicz A., Czyz M. (2019). Role of miRNAs in melanoma metastasis. Cancers.

[110] Xiao D., Barry S., Kmetz D., Egger M., Pan J., Rai S.N., Qu J., McMasters K.M., Hao H. (2016). Melanoma cell-derived exosomes promote epithelial-mesenchymal transition in primary melanocytes through paracrine/autocrine signaling in the tumor microenvironment. Cancer Lett..

[111] Pfeffer S.R., Grossmann K.F., Cassidy P.B., Yang C.H., Fan M., Kopelovich L., Leachman S.A., Pfeffer L.M. (2015). Detection of exosomal miRNAs in the plasma of melanoma patients. J. Clin. Med..

[112] Saldanha G., Potter L., Shendge P., Osborne J., Nicholson S., Yii N., Varma S., Aslam M.I., Elshaw S., Papadogeorgakis E. (2013). Plasma microRNA-21 is associated with tumor burden in cutaneous melanoma. J. Investig. Dermatol..

[113] Ragusa M., Barbagallo C., Statello L., Caltabiano R., Russo A., Puzzo L., Avitabile T., Longo A., Toro M.D., Barbagallo D. (2015). miRNA profiling in vitreous humor, vitreal exosomes and serum from uveal melanoma patients: Pathological and diagnostic implications. Cancer Biol. Ther..

[114] Korabiowska M., Dengler H., Kellner S., Stachura J., Schauer A. (1997). Decreased expression of MLH1, MSH2, PMS1 and PMS2 in pigmented lesions indicates accumulation of failed DNA repair along with malignant transformation and tumour progression. Oncol. Rep..

[115] Zhang Y., Chen Z., Feng L., Jiang P., Li X., Wang X. (2019). Ionizing radiation-inducible microRNA-21 induces angiogenesis by directly targeting PTEN. Asian Pac. J. Cancer Prev..

[116] Gabriely G., Wurdinger T., Kesari S., Esau C.C., Burchard J., Linsley P.S., Krichevsky A.M. (2008). MicroRNA 21 promotes glioma invasion by targeting matrix metalloproteinase regulators. Mol. Cell Biol..

[117] Liu L.Z., Li C., Chen Q., Jing Y., Carpenter R., Jiang Y., Kung H.F., Lai L., Jiang B.H. (2011). MiR-21 induced angiogenesis through AKT and ERK activation and HIF-1α expression. PLoS ONE.

[118] Hermansen S.K., Nielsen B.S., Aaberg-Jessen C., Kristensen B.W. (2016). miR-21 is linked to glioma angiogenesis: A co-localization study. J. Histochem. Cytochem..

[119] Du X., Hong L., Sun L., Sang H., Qian A., Li W., Zhuang H., Liang H., Song D., Li C. (2019). miR-21 induces endothelial progenitor cells proliferation and angiogenesis via targeting FASLG and is a potential prognostic marker in deep venous thrombosis. J. Transl. Med..

[120] Chen L.Y., Wang X., Qu X.L., Pan L.N., Wang Z.Y., Lu Y.H., Hu H.Y. (2019). Activation of the STAT3/microRNA-21 pathway participates in angiotensin II-induced angiogenesis. J. Cell Physiol..

[121] O′reilly A., Larkin J. (2017). Checkpoint inhibitors in advanced melanoma: Effect on the field of immunotherapy. Expert Rev. Anticancer Ther..

[122] Furue M., Ito T., Wada N., Wada M., Kadono T., Uchi H. (2018). Melanoma and immune checkpoint inhibitors. Curr. Oncol. Rep..

[123] Tokunaga A., Sugiyama D., Maeda Y., Warner A.B., Panageas K.S., Ito S., Togashi Y., Sakai C., Wolchok J.D., Nishikawa H. (2019). Selective inhibition of low-affinity memory CD8+ T cells by corticosteroids. J. Exp. Med..

[124] Molodtsov A., Turk M.J. (2018). Tissue resident CD8 memory T cell responses in cancer and autoimmunity. Front. Immunol..

[125] Malik B.T., Byrne K.T., Vella J.L., Zhang P., Shabaneh T.B., Steinberg S.M., Molodtsov A.K., Bowers J.S., Angeles C.V., Paulos C.M. (2017). Resident memory T cells in the skin mediate durable immunity to melanoma. Sci. Immunol..

[126] Willemsen M., Linkutė R., Luiten R.M., Matos T.R. (2019). Skin-resident memory T cells as a potential new therapeutic target in vitiligo and melanoma. Pigment Cell Melanoma Res..

[127] Massi D., Rulli E., Cossa M., Valeri B., Rodolfo M., Merelli B., De Logu F., Nassini R., Del Vecchio M., Di Guardo L. (2019). The density and spatial tissue distribution of CD8+ and CD163+ immune cells predict response and outcome in melanoma patients receiving MAPK inhibitors. J. Immunother. Cancer.

[128] Kim C., Hu B., Jadhav R.R., Jin J., Zhang H., Cavanagh M.M., Akondy R.S., Ahmed R., Weyand C.M., Goronzy J.J. (2018). Activation of miR-21-regulated pathways in immune aging selects against signatures characteristic of memory T Cells. Cell Rep..

[129] Sahraei M., Chaube B., Liu Y., Sun J., Kaplan A., Price N.L., Ding W., Oyaghire S., García-Milian R., Mehta S. (2019). Suppressing miR-21 activity in tumor-associated macrophages promotes an antitumor immune response. J. Clin. Investig..

[130] Mathsyaraja H., Thies K., Taffany D.A., Deighan C., Liu T., Yu L., Fernandez S.A., Shapiro C., Otero J., Timmers C. (2015). CSF1-ETS2-induced microRNA in myeloid cells promote metastatic tumor growth. Oncogene.

[131] Wolf S.F., Sieburth D., Sypek J. (1994). Interleukin 12: A key modulator of immune function. Stem Cells.

[132] Lu X. (2017). Impact of IL-12 in cancer. Curr. Cancer Drug Targets.

[133] Cocco C., Pistoia V., Airoldi I. (2009). New perspectives for melanoma immunotherapy: Role of IL-12. Curr. Mol. Med..

[134] Nagai H., Oniki S., Fujiwara S., Yoshimoto T., Nishigori C. (2010). Antimelanoma immunotherapy: Clinical and preclinical applications of IL-12 family members. Immunotherapy.

[135] Tucci M., Mannavola F., Passarelli A., Stucci L.S., Cives M., Silvestris F. (2018). Exosomes in melanoma: A role in tumor progression, metastasis and impaired immune system activity. Oncotarget.

[136] Sharma P., Diergaarde B., Ferrone S., Kirkwood J.M., Whiteside T.L. (2020). Melanoma cell-derived exosomes in plasma of melanoma patients suppress functions of immune effector cells. Sci. Rep..

[137] Cordonnier M., Nardin C., Chanteloup G., Derangere V., Algros M.P., Arnould L., Garrido C., Aubin F., Gobbo J. (2020). Tracking the evolution of circulating exosomal-PD-L1 to monitor melanoma patients. J. Extracell. Vesicles.

[138] Vignard V., Labbé M., Marec N., André-Grégoire G., Jouand N., Fonteneau J.F., Labarrière N., Fradin D. (2020). MicroRNAs in tumor exosomes drive immune escape in melanoma. Cancer Immunol. Res..

[139] Yang C.H., Yue J., Pfeffer S.R., Fan M., Paulus E., Hosni-Ahmed A., Sims M., Qayyum S., Davidoff A.M., Handorf C.R. (2014). MicroRNA-21 promotes glioblastoma tumorigenesis by down-regulating insulin-like growth factor-binding protein-3 (IGFBP3). J. Biol. Chem..

[140] Masoudi M.S., Mehrabian E., Mirzaei H. (2018). MiR-21: A key player in glioblastoma pathogenesis. J. Cell. Biochem..

[141] Ribas J., Lupold S.E. (2010). The transcriptional regulation of miR-21, its multiple transcripts, and their implication in prostate cancer. Cell Cycle.

[142] Guan C., Zhang L., Wang S., Long L., Zhou H., Qian S., Ma M., Bai F., Meng Q.H., Lyu J. (2019). Upregulation of MicroRNA-21 promotes tumorigenesis of prostate cancer cells by targeting KLF5. Cancer Biol. Ther..

[143] Scarbrough P.M., Akushevich I., Wrensch M., Il′yasova D. (2014). Exploring the association between melanoma and glioma risks. Ann. Epidemiol..

[144] Patasius A., Urbonas V., Smailyte G. (2019). Skin melanoma and subsequent risk of prostate cancer: A Lithuanian Cancer Registry Study. Int. J. Environ. Res. Public Health.

[145] Cole-Clark D., Nair-Shalliker V., Bang A., Rasiah K., Chalasani V., Smith D.P. (2018). An initial melanoma diagnosis may increase the subsequent risk of prostate cancer: Results from the New South Wales Cancer Registry. Sci. Rep..

[146] Sutcliffe S., Giovannucci E., Isaacs W.B., Willett W.C., Platz E.A. (2007). Acne and risk of prostate cancer. Int. J. Cancer.

[147] Ugge H., Udumyan R., Carlsson J., Andrén O., Montgomery S., Davidsson S., Fall K. (2018). Acne in late adolescence and risk of prostate cancer. Int. J. Cancer.

[148] Zhang M., Qureshi A.A., Fortner R.T., Hankinson S.E., Wei Q., Wang L.E., Eliassen A.H., Willett W.C., Hunter D.J., Han J. (2015). Teenage acne and cancer risk in US women: A prospective cohort study. Cancer.

[149] Mota Garcia T., Hiyoshi A., Udumyan R., Sjöqvist H., Fall K., Montgomery S. (2017). Acne in late adolescence is not associated with a raised risk of subsequent malignant melanoma among men. Cancer Epidemiol..

[150] Dréno B., Bettoli V., Araviiskaia E., Sanchez Viera M., Bouloc A. (2018). The influence of exposome on acne. J. Eur. Acad. Dermatol. Venereol..

[151] Melnik B.C. (2015). MiR-21: An environmental driver of malignant melanoma?. J. Transl. Med..

[152] Puckett Y., Wilson A.M., Thevenin C. (2020). Cancer, Melanoma Pathology.

[153] Sample A., He Y.Y. (2018). Mechanisms and prevention of UV-induced melanoma. Photodermatol. Photoimmunol. Photomed..

[154] Ellerhorst J.A., Greene V.R., Ekmekcioglu S., Warneke C.L., Johnson M.M., Cooke C.P., Wang L.E., Prieto V.G., Gershenwald J.E., Wei Q. (2011). Clinical correlates of NRAS and BRAF mutations in primary human melanoma. Clin. Cancer Res..

[155] Curtin J.A., Fridlyand J., Kageshita T., Patel H.N., Busam K.J., Kutzner H., Cho K.H., Aiba S., Bröcker E.B., LeBoit P.E. (2005). Distinct sets of genetic alterations in melanoma. N. Engl. J. Med..

[156] Maldonado J.L., Fridlyand J., Patel H., Jain A.N., Busam K., Kageshita T., Ono T., Albertson D.G., Pinkel D., Bastian B.C. (2003). Determinants of BRAF mutations in primary melanomas. J. Natl. Cancer Inst..

[157] Edwards R.H., Ward M.R., Wu H., Medina C.A., Brose M.S., Volpe P., Nussen-Lee S., Haupt H.M., Martin A.M., Herlyn M. (2004). Absence of BRAF mutations in UV-protected mucosal melanomas. J. Med. Genet..

[158] Bauer J., Büttner P., Murali R., Okamoto I., Kolaitis N.A., Landi M.T., Scolder R.A., Bastian B.C. (2011). BRAF mutations in cutaneous melanoma are independently associated with age, anatomic site of the primary tumor, and the degree of solar elastosis at the primary tumor site. Pigment Cell Melanoma Res..

[159] Whiteman D.C., Stickle M., Watt P., Hughes M.C., Davis M.B., Green A.C. (2006). Anatomic site, sun exposure, and risk of cutaneous melanoma. J. Clin. Oncol..

[160] Lo Cicero A., Delevoye C., Gilles-Marsens F., Loew D., Dingli F., Guéré C., André N., Vié K., van Niel G., Raposo G. (2015). Exosomes released by keratinocytes modulate melanocyte pigmentation. Nat. Commun..

[161] Liu Y., Xue L., Gao H., Chang L., Yu X., Zhu Z., He X., Geng J., Dong Y., Li H. (2019). Exosomal miRNA derived from keratinocytes regulates pigmentation in melanocytes. J. Dermatol. Sci..

[162] Wäster P., Eriksson I., Vainikka L., Öllinger K. (2020). Extracellular vesicles released by melanocytes after UVA irradiation promote intercellular signaling via miR21. Pigment Cell Melanoma Res..

[163] Syed D.N., Khan M.I., Shabbir M., Mukhtar H. (2013). MicroRNAs in skin response to UV radiation. Curr. Drug Targets.

[164] Hou L., Bowman L., Meighan T.G., Pratheeshkumar P., Shi X., Ding M. (2013). Induction of miR-21-PDCD4 signaling by UVB in JB6 cells involves ROS-mediated MAPK pathways. Exp. Toxicol. Pathol..

[165] Guo L., Huang Z.X., Chen X.W., Deng Q.K., Yan W., Zhou M.J., Ou C.S., Ding Z.H. (2009). Differential expression profiles of microRNAs in NIH3T3 cells in response to UVB irradiation. Photochem. Photobiol..

[166] Lin K.Y., Chen C.M., Lu C.Y., Cheng C.Y., Wu Y.H. (2017). Regulation of miR-21 expression in human melanoma via UV-ray-induced melanin pigmentation. Environ. Toxicol..

[167] Degueurce G., D′Errico I., Pich C., Ibberson M., Schütz F., Montagner A., Sgandurra M., Mury L., Jafari P., Boda A. (2016). Identification of a novel PPARβ/δ/miR-21-3p axis in UV-induced skin inflammation. EMBO Mol. Med..

[168] Hartman M.L., Czyz M. (2015). MITF in melanoma: Mechanisms behind its expression and activity. Cell Mol. Life Sci..

[169] Fishel R., Ewel A., Lee S., Lescoe M.K., Griffith J. (1994). Binding of mismatched microsatellite DNA sequences by the human MSH2 protein. Science.

[170] Kubeček O., Kopecký J. (2016). Microsatellite instability in melanoma: A comprehensive review. Melanoma Res..

[171] Korabiowska M., König F., Verheggen R., Schlott T., Cordon-Cardo C., Romeike B., Brinck U. (2004). Altered expression and new mutations in DNA mismatch repair genes MLH1 and MSH2 in melanoma brain metastases. Anticancer Res..

[172] Korabiowska M., Brinck U., Stachura J., Jawien J., Hasse F.M., Cordon-Cardos C., Fischer G. (2006). Exonic deletions of mismatch repair genes MLH1 and MSH2 correlate with prognosis and protein expression levels in malignant melanomas. Anticancer Res..

[173] Hussein M.R., Wood G.S. (2003). hMLH1 and hMSH2 gene mutations are present in radial growth-phase cutaneous malignant melanoma cell lines and can be induced further by ultraviolet-B irradiation. Exp. Dermatol..

[174] Sanlorenzo M., Wehner M.R., Linos E., Kornak J., Kainz W., Posch C., Vujic I., Johnston K., Gho D., Monico G. (2015). The risk of melanoma in airline pilots and cabin crew: A meta-analysis. JAMA Dermatol..

[175] Miura K., Olsen C.M., Rea S., Marsden J., Green A.C. (2019). Do airline pilots and cabin crew have raised risks of melanoma and other skin cancers? Systematic review and meta-analysis. Br. J. Dermatol..

[176] Wang J., Zhang X., Wang P., Wang X., Farris A.B., Wang Y. (2016). Lessons learned using different mouse models during space radiation-induced lung tumorigenesis experiments. Life Sci. Space Res..

[177] Shi Y., Zhang X., Tang X., Wang P., Wang H., Wang Y. (2012). MiR-21 is continually elevated long-term in the brain after exposure to ionizing radiation. Radiat. Res..

[178] Zhu Y., Yu X., Fu H., Wang H., Wang P., Zheng X., Wang Y. (2010). MicroRNA-21 is involved in ionizing radiation-promoted liver carcinogenesis. Int. J. Clin. Exp. Med..

[179] Liu Z., Liang X., Li X., Liu X., Zhu M., Gu Y., Zhou P. (2019). MiRNA-21 functions in ionizing radiation-induced epithelium-to-mesenchymal transition (EMT) by downregulating PTEN. Toxicol. Res..

[180] Xu S., Ding N., Pei H., Hu W., Wie W., Zhang X., Zhou G., Wang J. (2014). MiR-21 is involved in radiation-induced bystander effects. RNA Biol..

[181] Xu S., Wang J., Ding N., Hu W., Zhang X., Wang B., Hua J., Wie W., Zhu Q. (2015). Exosome-mediated microRNA transfer plays a role in radiation-induced bystander effect. RNA Biol..

[182] Nicholas J.S., Lackland D.T., Butler G.C., Mohr L.C., Dunbar J.B., Kaune W.T., Grosche B., Hoel D.G. (1998). Cosmic radiation and magnetic field exposure to airline flight crews. Am. J. Ind. Med..

[183] Selvamurugan N., He Z., Rifkin D., Dabovic B., Partridge N.C. (2017). Pulsed electromagnetic field regulates microRNA 21 expression to activate TGF-β signaling in human bone marrow stromal cells to enhance osteoblast differentiation. Stem Cells Int..

[184] Sanlorenzo M., Vujic I., Posch C., Cleaver J.E., Quaglino P., Ortiz-Urda S. (2015). The risk of melanoma in pilots and cabin crew: UV measurements in flying airplanes. JAMA Dermatol..

[185] Hallberg Ö. (2016). Cancer incidence vs. FM radio transmitter density. Electromagn. Biol. Med..

[186] Irvine D., Davies D.M. (1992). The mortality of British Airways pilots, 1966–1989: A proportional mortality study. Aviat. Space Environ. Med..

[187] Yong L.C., Pinkerton L.E., Yiin J.H., Anderson J.L., Deddens J.A. (2014). Mortality among a cohort of U.S. commercial airline cockpit crew. Am. J. Ind. Med..

[188] Dupin E., Real C., Glavieux-Pardanaud C., Vaigot P., Le Douarin N.M. (2003). Reversal of developmental restrictions in neural crest lineages: Transition from Schwann cells to glial-melanocytic precursors in vitro. Proc. Natl. Acad. Sci. USA.

[189] Yang M., Guo W., Yang C., Tang J., Huang Q., Feng S., Jiang A., Xu X., Jiang G. (2017). Mobile phone use and glioma risk: A systematic review and meta-analysis. PLoS ONE.

[190] Morgan L.L., Miller A.B., Sasco A., Davis D.L. (2015). Mobile phone radiation causes brain tumors and should be classified as a probable human carcinogen (2A) (review). Int. J. Oncol..

[191] Poulsen A.H., Friis S., Johansen C., Jensen A., Frei P., Kjaear S.K., Dalton S.O., Schüz J. (2013). Mobile phone use and the risk of skin cancer: A nationwide cohort study in Denmark. Am. J. Epidemiol..

[192] Zeller J., Strack C., Fenk S., Mohr M., Loew T., Schmitz G., Maier L., Fischer M., Baessler A. (2016). Relation between obesity, metabolic syndrome, successful long-term weight reduction, and right ventricular function. Int. Heart J..

[193] Sherling D.H., Perumareddi P., Hennekens C.H. (2017). Metabolic syndrome. J. Cardiovasc. Pharmacol. Ther..

[194] Hoch D., Gauster M., Hauguel-de Mouzon S., Desoye G. (2019). Diabesity-associated oxidative and inflammatory stress signalling in the early human placenta. Mol. Aspects Med..

[195] Alarcón S., Niechi I., Toledo F., Sobrevia L., Quezada C. (2019). Glioma progression in diabesity. Mol. Aspects Med..

[196] Yu Z.B., Han S.P., Zhu G.Z., Zhu C., Wang X.J., Cao X.G., Guo X.R. (2011). Birth weight and subsequent risk of obesity: A systematic review and meta-analysis. Obes. Rev..

[197] Werneck A.O., Silva D.R.P., Collings P.J., Fernandes R.A., Ronque E.R.V., Coelho-E-Silva M.J., Sardinha L.B., Cyrino E.S. (2017). Birth weight, biological maturation and obesity in adolescents: A mediation analysis. J. Dev. Orig. Health Dis..

[198] Knop M.R., Geng T.T., Gorny A.W., Ding R., Li C., Ley S.H., Huang T. (2018). Birth weight and risk of type 2 diabetes mellitus, cardiovascular disease, and hypertension in adults: A meta-analysis of 7 646 267 participants from 135 studies. J. Am. Heart Assoc..

[199] Ward Z.J., Long M.W., Resch S.C., Giles C.M., Cradock A.L., Gortmaker S.L. (2017). Simulation of growth trajectories of childhood obesity into adulthood. N. Engl. J. Med..

[200] Salihu H.M., Dongarwar D., King L.M., Yusuf K.K., Ibrahimi S., Salinas-Miranda A.A. (2020). Trends in the incidence of fetal macrosomia and its phenotypes in the United States, 1971-2017. Arch. Gynecol. Obstet..

[201] Hermann G.M., Dallas L.M., Haskell S.E., Roghair R.D. (2010). Neonatal macrosomia is an independent risk factor for adult metabolic syndrome. Neonatology.

[202] Wojcik K.Y., Escobedo L.A., Wysong A., Heck J.E., Ritz B., Hamilton A.S., Milam J., Cockburn M.G. (2019). High birth weight, early UV exposure, and melanoma risk in children, adolescents, and young adults. Epidemiology.

[203] Jiang H., Wu W., Zhang M., Li J., Peng Y., Miao T.T., Zhu H., Xu G. (2014). Aberrant upregulation of miR-21 in placental tissues of macrosomia. J. Perinatol..

[204] Zhang J.T., Cai Q.Y., Ji S.S., Zhang H.X., Wang Y.H., Yan H.T., Yang X.J. (2016). Decreased miR-143 and increased miR-21 placental expression levels are associated with macrosomia. Mol. Med. Rep..

[205] Mitchell M.D., Peiris H.N., Kobayashi M., Koh Y.Q., Duncombe G., Illanes S.E., Rice G.E., Salomon C. (2015). Placental exosomes in normal and complicated pregnancy. Am. J. Obstet. Gynecol..

[206] Pillay P., Moodley K., Moodley J., Mackraj I. (2017). Placenta-derived exosomes: Potential biomarkers of preeclampsia. Int. J. Nanomedicine.

[207] Meyle K.D., Gamborg M., Sørensen T.I.A., Baker J.L. (2017). Childhood body size and the risk of malignant melanoma in adulthood. Am. J. Epidemiol..

[208] Dusingize J.C., Olsen C.M., An J., Pandeya N., Law M.H., Thompson B.S., Goldstein A.M., Iles M.M., Webb P.M., Neale R.E. (2020). Body mass index and height and risk of cutaneous melanoma: Mendelian randomization analyses. Int. J. Epidemiol..

[209] Hoppe C., Mølgaard C., Michaelsen K.F. (2006). Cow′s milk and linear growth in industrialized and developing countries. Annu. Rev. Nutr..

[210] Chen X., Gao C., Li H., Huang L., Sun Q., Dong Y., Tian C., Gao S., Dong H., Guan D. (2010). Identification and characterization of microRNAs in raw milk during different periods of lactation, commercial fluid, and powdered milk products. Cell Res..

[211] Melnik B.C. (2015). Milk—A nutrient system of mammalian evolution promoting mTORC1-dependent translation. Int. J. Mol. Sci..

[212] Yu S., Zhao Z., Sun L., Li P. (2017). Fermentation results in quantitative changes in milk-derived exosomes and different effects on cell growth and survival. J. Agric. Food Chem..

[213] Melnik B.C., Schmitz G. (2019). Exosomes of pasteurized milk: Potential pathogens of Western diseases. J. Transl. Med..

[214] Melnik B.C. (2016). Western diet-induced imbalances of FoxO1 and mTORC1 signalling promote the sebofollicular inflammasomopathy acne vulgaris. Exp. Dermatol..

[215] Agamia N.F., Abdallah D.M., Sorour O., Mourad B., Younan D.N. (2016). Skin expression of mammalian target of rapamycin and forkhead box transcription factor O1, and serum insulin-like growth factor-1 in patients with acne vulgaris and their relationship with diet. Br. J. Dermatol..

[216] Bai L., Liang R., Yang Y., Hou X., Wang Z., Zhu S., Wang C., Tang Z., Li K. (2015). MicroRNA-21 regulates PI3K/Akt/mTOR signaling by targeting TGFβI during skeletal muscle development in pigs. PLoS ONE.

[217] Yang C., Liu X., Zhao K., Zhu Y., Hu B., Zhou Y., Wang M., Wu Y., Zhang C., Xu J. (2019). MiRNA-21 promotes osteogenesis via the PTEN/PI3K/Akt/HIF-1α pathway and enhances bone regeneration in critical size defects. Stem Cell Res. Ther..

[218] Damsky W., Micevic G., Meeth K., Muthusamy V., Curley D.P., Santhanakrishnan M., Erdelyi I., Platt J.T., Huang L., Theodosakis N. (2015). mTORC1 activation blocks BrafV600E-induced growth arrest but is insufficient for melanoma formation. Cancer Cell.

[219] Dey N., Das F., Ghosh-Choudhury N., Mandal C.C., Parekh D.J., Block K., Kasinath B.S., Abboud H.E., Choudhury G.G. (2012). microRNA-21 governs TORC1 activation in renal cancer cell proliferation and invasion. PLoS ONE.

[220] Brandon E.L., Gu J.W., Cantwell L., He Z., Wallace G., Hall J.E. (2009). Obesity promotes melanoma tumor growth: Role of leptin. Cancer Biol. Ther..

[221] Pandey V., Vijayakumar M.V., Ajay A.K., Malvi P., Bhat M.K. (2012). Diet-induced obesity increases melanoma progression: Involvement of Cav-1 and FASN. Int. J. Cancer.

[222] Chen J., Chi M., Chen C., Zhang X.D. (2013). Obesity and melanoma: Exploring molecular links. J. Cell. Biochem..

[223] Malvi P., Chaube B., Pandey V., Vijayakumar M.V., Boreddy P.R., Mohammad N., Singh S.V., Bhat M.K. (2015). Obesity induced rapid melanoma progression is reversed by orlistat treatment and dietary intervention: Role of adipokines. Mol. Oncol..

[224] Karimi K., Lindgren T.H., Koch C.A., Brodell R.T. (2016). Obesity as a risk factor for malignant melanoma and non-melanoma skin cancer. Rev. Endocr. Metab. Disord..

[225] De Giorgi V., Gori A., Savarese I., D′Errico A., Scarfì F., Papi F., Maio V., Covarelli P., Massi D., Gandini S. (2017). Role of BMI and hormone therapy in melanoma risk: A case-control study. J. Cancer Res. Clin. Oncol..

[226] Coelho P., Almeida J., Prudêncio C., Fernandes R., Soares R. (2016). Effect of adipocyte secretome in melanoma progression and vasculogenic mimicry. J. Cell. Biochem..

[227] Ko J.H., Um J.Y., Lee S.G., Yang W.M., Sethi G., Ahn K.S. (2019). Conditioned media from adipocytes promote proliferation, migration, and invasion in melanoma and colorectal cancer cells. J. Cell. Physiol..

[228] Lazar I., Clement E., Dauvillier S., Milhas D., Ducoux-Petit M., LeGonidec S., Moro C., Soldan V., Dalle S., Balor S. (2016). Adipocyte exosomes promote melanoma aggressiveness through fatty acid oxidation: A novel mechanism linking obesity and cancer. Cancer Res..

[229] Clement E., Lazar I., Muller C., Nieto L. (2017). Obesity and melanoma: Could fat be fueling malignancy?. Pigment Cell Melanoma Res..

[230] Kim Y.J., Hwang S.H., Cho H.H., Shin K.K., Bae Y.C., Jung J.S. (2012). MicroRNA 21 regulates the proliferation of human adipose tissue-derived mesenchymal stem cells and high-fat diet-induced obesity alters microRNA 21 expression in white adipose tissues. J. Cell. Physiol..

[231] Chartoumpekis D.V., Zaravinos A., Ziros P.G., Iskrenova R.P., Psyrogiannis A.I., Kyriazopoulou V.E., Habeos I.G. (2012). Differential expression of microRNAs in adipose tissue after long-term high-fat diet-induced obesity in mice. PLoS ONE.

[232] An Y., Zhao J., Nie F., Qin Z., Xue H., Wang G., Li D. (2019). Exosomes from adipose-derived stem cells (ADSCs) overexpressing miR-21 promote vascularization of endothelial cells. Sci. Rep..

[233] Yang C., Luo L., Bai X., Shen K., Liu K., Wang J., Hu D. (2020). Highly-expressed micoRNA-21 in adipose derived stem cell exosomes can enhance the migration and proliferation of the HaCaT cells by increasing the MMP-9 expression through the PI3K/AKT pathway. Arch. Biochem. Biophys..

[234] Amjadi F., Javanmard S.H., Zarkesh-Esfahani H., Khazaei M., Narimani M. (2011). Leptin promotes melanoma tumor growth in mice related to increasing circulating endothelial progenitor cells numbers and plasma NO production. J. Exp. Clin. Cancer.

[235] Park H.K., Ahima R.S. (2014). Leptin signaling. F1000Prime Rep..

[236] Zhang N., Zhang N., Song L., Xie H., Zhao C., Li S., Zhao W., Zhao Y., Gao C., Xu G. (2017). Adipokines and free fatty acids regulate insulin sensitivity by increasing microRNA-21 expression in human mature adipocytes. Mol. Med. Rep..

[237] Nieman K.M., Romero I.L., Van Houten B., Lengyel E. (2013). Adipose tissue and adipocytes support tumorigenesis and metastasis. Biochim. Biophys. Acta.

[238] Au Yeung C.L., Co N.N., Tsuruga T., Yeung T.L., Kwan S.Y., Leung C.S., Li Y., Lu E.S., Kwan K., Wong K.K. (2016). Exosomal transfer of stroma-derived miR21 confers paclitaxel resistance in ovarian cancer cells through targeting APAF1. Nat. Commun..

[239] Qi L., Qi X., Xiong H., Liu Q., Li J., Zhang Y., Ma X., Wu N., Liu Q., Feng L. (2014). Type 2 diabetes mellitus and risk of malignant melanoma: A systematic review and meta-analysis of cohort studies. Iran J. Public Health.

[240] Sekar D., Venugopal B., Sekar P., Ramalingam K. (2016). Role of microRNA 21 in diabetes and associated/related diseases. Gene.

[241] Nunez Lopez Y.O., Garufi G., Seyhan A.A. (2016). Altered levels of circulating cytokines and microRNAs in lean and obese individuals with prediabetes and type 2 diabetes. Mol. Biosyst..

[242] Seyhan A.A., Nunez Lopez Y.O., Xie H., Yi F., Mathews C., Pasarica M., Pratley R.E. (2016). Pancreas-enriched miRNAs are altered in the circulation of subjects with diabetes: A pilot cross-sectional study. Sci. Rep..

[243] Wu H., Kong L., Tan Y., Epstein P.N., Zeng J., Gu J., Liang G., Kong M., Chen X., Miao L. (2016). C66 ameliorates diabetic nephropathy in mice by both upregulating NRF2 function via increase in miR-200a and inhibiting miR-21. Diabetologia.

[244] Lakhter A.J., Pratt R.E., Moore R.E., Doucette K.K., Maier B.F., DiMeglio L.A., Sims E.K. (2018). Beta cell extracellular vesicle miR-21-5p cargo is increased in response to inflammatory cytokines and serves as a biomarker of type 1 diabetes. Diabetologia.

[245] Stocks T., Van Hemelrijck M., Manjer J., Bjørge T., Ulmer H., Hallmans G., Lindkvist B., Selmer R., Nagel G., Tretli S. (2012). Blood pressure and risk of cancer incidence and mortality in the Metabolic Syndrome and Cancer Project. Hypertension.

[246] Nagel G., Bjørge T., Stocks T., Manjer J., Hallmans G., Edlinger M., Häggström C., Engeland A., Johansen D., Kleiner A. (2012). Metabolic risk factors and skin cancer in the Metabolic Syndrome and Cancer Project (Me-Can). Br. J. Dermatol..

[247] Radišauskas R., Kuzmickienė I., Milinavičienė E., Everatt R. (2016). Hypertension, serum lipids and cancer risk: A review of epidemiological evidence. Medicina.

[248] Warner A.B., McQuade J.L. (2019). Modifiable host factors in melanoma: Emerging evidence for obesity, diet, exercise, and the microbiome. Curr. Oncol. Rep..

[249] Hall K.D. (2018). Did the food environment cause the obesity epidemic?. Obesity.

[250] Chen G.L., Luo Y., Eriksson D., Meng X., Qian C., Bäuerle T., Chen X.X., Schett G., Bozec A. (2016). High fat diet increases melanoma cell growth in the bone marrow by inducing osteopontin and interleukin 6. Oncotarget.

[251] Malagoli C., Malavolti M., Farnetani F., Longo C., Filippini T., Pellacani G., Vinceti M. (2019). Food and beverage consumption and melanoma risk: A population-based case-control study in northern Italy. Nutrients.

[252] Malavolti M., Malagoli C., Crespi C.M., Brighenti F., Agnoli C., Sieri S., Krogh V., Fiorentini C., Farnetani F., Longo C. (2017). Glycaemic index, glycaemic load and risk of cutaneous melanoma in a population-based, case-control study. Br. J. Nutr..

[253] Burris J., Shikany J.M., Rietkerk W., Woolf K. (2018). A low glycemic index and glycemic load diet decreases insulin-like growth factor-1 among adults with moderate and severe acne: A short-duration, 2-week randomized controlled trial. J. Acad. Nutr. Diet.

[254] Melnik B. (2012). Dietary intervention in acne: Attenuation of increased mTORC1 signaling promoted by Western diet. Dermatoendocrinology.

[255] Zeng J., Xiong Y., Li G., Liu M., He T., Tang Y., Chen Y., Cai L., Jiang R., Tao J. (2013). MiR-21 is overexpressed in response to high glucose and protects endothelial cells from apoptosis. Exp. Clin. Endocrinol. Diabetes.

[256] Hanousková B., Neprašová B., Skálová L., Maletínská L., Zemanová K., Ambrož M., Matoušková P. (2019). High-fructose drinks affect microRNAs expression differently in lean and obese mice. J. Nutr. Biochem..

[257] Castellana M., Conte E., Cignarelli A., Perrini S., Giustina A., Giovanella L., Giorgino F., Trimboli P. (2020). Efficacy and safety of very low calorie ketogenic diet (VLCKD) in patients with overweight and obesity: A systematic review and meta-analysis. Rev. Endocr. Metab. Disord..

[258] McDonald T.J.W., Cervenka M.C. (2019). Lessons learned from recent clinical trials of ketogenic diet therapies in adults. Curr. Opin. Clin. Nutr. Metab. Care.

[259] Xia S., Lin R., Jin L., Zhao L., Kang H.B., Pan Y., Liu S., Qian G., Qian Z., Konstantakou E. (2017). Prevention of dietary-fat-fueled ketogenesis attenuates BRAF V600E tumor growth. Cell Metab..

[260] Kang H.B., Fan J., Lin R., Elf S., Ji Q., Zhao L., Jin L., Seo J.H., Shan C., Arbiser J.L. (2015). Metabolic rewiring by oncogenic BRAF V600E links ketogenesis pathway to BRAF-MEK1 signaling. Mol. Cell..

[261] Zhao L., Fan J., Xia S., Pan Y., Liu S., Qian G., Qian Z., Kang H.B., Arbiser J.L., Pollack B.P. (2017). HMG-CoA synthase 1 is a synthetic lethal partner of BRAFV600E in human cancers. J. Biol. Chem..

[262] Pratilas C.A., Taylor B.S., Ye Q., Viale A., Sander C., Solit D.B., Rosen N. (2009). (V600E) BRAF is associated with disabled feedback inhibition of RAF-MEK signaling and elevated transcriptional output of the pathway. Proc. Natl. Acad. Sci. USA.

[263] Banikazemi Z., Haji H.A., Mohammadi M., Taheripak G., Iranifar E., Poursadeghiyan M., Moridikia A., Rashidi B., Taghizadeh M., Mirzaei H. (2018). Diet and cancer prevention: Dietary compounds, dietary microRNAs, and dietary exosomes. J. Cell. Biochem..

[264] Munir J., Lee M., Ryu S. (2020). Exosomes in food: Health benefits and clinical relevance in diseases. Adv. Nutr..

[265] Manca S., Upadhyaya B., Mutai E., Desaulniers A.T., Cederberg R.A., White B.R., Zempleni J. (2018). Milk exosomes are bioavailable and distinct microRNA cargos have unique tissue distribution patterns. Sci. Rep..

[266] Zempleni J., Sukreet S., Zhou F., Wu D., Mutai E. (2019). Milk-derived exosomes and metabolic regulation. Annu. Rev. Anim. Biosci..

[267] Golan-Gerstl R., Elbaum Shiff Y., Moshayoff V., Schecter D., Leshkowitz D., Reif S. (2017). Characterization and biological function of milk-derived miRNAs. Mol. Nutr. Food Res..

[268] Tucker L.A. (2019). Milk fat intake and telomere length in U.S. women and men: The role of the milk fat fraction. Oxid. Med. Cell. Longev..

[269] Zhu H.Y., Li C., Bai W.D., Su L.L., Liu J.Q., Li Y., Shi J.H., Cai W.X., Bai X.Z., Jia Y.H. (2014). MicroRNA-21 regulates hTERT via PTEN in hypertrophic scar fibroblasts. PLoS ONE.

[270] Yang Y., Yang J.J., Tao H., Jin W.S. (2015). MicroRNA-21 controls hTERT via PTEN in human colorectal cancer cell proliferation. J. Physiol. Biochem..

[271] De Unamuno Bustos B., Sahuquillo Torralba A., Moles Poveda P., Pérez Simó G., Simarro Farinos J., Llavador Ros M., Palanca Suela S., Botella Estrada R. (2019). Telomerase expression in a series of melanocytic neoplasms. Actas Dermosifiliogr..

[272] Shaughnessy M., Njauw C.N., Artomov M., Tsao H. (2020). Classifying melanoma by TERT promoter mutational status. J. Investig. Dermatol..

[273] Grant B.F., Chou S.P., Saha T.D., Pickering R.P., Kerridge B.T., Ruan W.J., Huang B., Jung J., Zhang H., Fan A. (2017). Prevalence of 12-month alcohol use, high-risk drinking, and DSM-IV alcohol use disorder in the United States, 2001–2002 to 2012–2013: Results from the National Epidemiologic Survey on Alcohol and Related Conditions. JAMA Psychiatry.

[274] Yang K., Fung T.T., Nan H. (2018). An epidemiological review of diet and cutaneous malignant melanoma. Cancer Epidemiol. Biomarkers Prev..

[275] Lefèvre A., Adler H., Lieber C.S. (1970). Effect of ethanol on ketone metabolism. J. Clin. Investig..

[276] Beech R.D., Leffert J.J., Lin A., Hong K.A., Hansen J., Umlauf S., Mane S., Zhao H., Sinha R. (2014). Stress-related alcohol consumption in heavy drinkers correlates with expression of miR-10a, miR-21, and components of the TAR-RNA-binding protein-associated complex. Alcohol Clin. Exp. Res..

[277] Bian J.T., Piano M.R., Kotlo K.U., Mahmoud A.M., Phillips S.A. (2018). MicroRNA-21 contributes to reduced microvascular function in binge drinking young adults. Alcohol Clin. Exp. Res..

[278] Donat-Vargas C., Berglund M., Glynn A., Wolk A., Åkesson A. (2017). Dietary polychlorinated biphenyls, long-chain n-3 polyunsaturated fatty acids and incidence of malignant melanoma. Eur. J. Cancer.

[279] Ju L., Zhou Z., Jiang B., Lou Y., Zhang Z. (2017). miR-21 is involved in skeletal deficiencies of zebrafish embryos exposed to polychlorinated biphenyls. Environ. Sci. Pollut. Res. Int..

[280] Wahlang B., Petriello M.C., Perkins J.T., Shen S., Hennig B. (2016). Polychlorinated biphenyl exposure alters the expression profile of microRNAs associated with vascular diseases. Toxicol. In Vitro.

[281] Furman D., Campisi J., Verdin E., Carrera-Bastos P., Targ S., Franceschi C., Ferrucci L., Gilroy D.W., Fasano A., Miller G.W. (2019). Chronic inflammation in the etiology of disease across the life span. Nat. Med..

[282] Newton-Bishop J.A., Davies J.R., Latheef F., Randerson-Moor J., Chan M., Gascoyne J., Waseem S., Haynes S., O′Donovan C., Bishop D.T. (2015). 25-Hydroxyvitamin D2/D3 levels and factors associated with systemic inflammation and melanoma survival in the Leeds Melanoma Cohort. Int. J. Cancer.

[283] Hardie C.M., Elliott F., Chan M., Rogers Z., Bishop D.T., Newton-Bishop J.A. (2020). Environmental exposures such as smoking and low vitamin D are predictive of poor outcome in cutaneous melanoma rather than other deprivation measures. J. Investig. Dermatol..

[284] Dusingize J.C., Olsen C.M., Pandeya N., Thompson B.S., Webb P.M., Green A.C., Neale R.E., Whiteman D.C. (2018). QSkin Study. Smoking and cutaneous melanoma: Findings from the QSkin Sun and Health Cohort Study. Cancer Epidemiol. Biomarkers Prev..

[285] Gibson J.A.G., Dobbs T.D., Griffiths R., Song J., Akbari A., Whitaker S., Watkins A., Langan S.M., Hutchings H.A., Lyons R.A. (2020). The association of smoking and socioeconomic status on cutaneous melanoma: A population based, data linkage, case-control study. Br. J. Dermatol..

[286] Sondermeijer L., Lamboo L.G.E., de Waal A.C., Galesloot T.E., Kiemeney L.A.L.M., van Rossum M., Aben K.H. (2020). Cigarette smoking and the risk of cutaneous melanoma: A case-control study. Dermatology.

[287] Zhang Y., Pan T., Zhong X., Cheng C. (2014). Nicotine upregulates microRNA-21 and promotes TGF-β-dependent epithelial-mesenchymal transition of esophageal cancer cells. Tumour Biol..

[288] Xu H., Ling M., Xue J., Dai X., Sun Q., Chen C., Liu Y., Zhou L., Liu J., Luo F. (2018). Exosomal microRNA-21 derived from bronchial epithelial cells is involved in aberrant epithelium-fibroblast cross-talk in COPD induced by cigarette smoking. Theranostics.

[289] Zhu J., Liu B., Wang Z., Wang D., Ni H., Zhang L., Wang Y. (2019). Exosomes from nicotine-stimulated macrophages accelerate atherosclerosis through miR-21-3p/PTEN-mediated VSMC migration and proliferation. Theranostics.

[290] Zhou F., Li S., Jia W., Lv G., Song C., Kang C., Zhang Q. (2015). Effects of diesel exhaust particles on microRNA-21 in human bronchial epithelial cells and potential carcinogenic mechanisms. Mol. Med. Rep..

[291] Goldenberg A., Jiang S.I., Cohen P.R. (2015). A possible association between melanoma and prostate cancer. Results from a case-control-study. Cancers.

[292] Wang Y., Ou Z., Sun Y., Yeh S., Wang X., Long J., Chang C. (2017). Androgen receptor promotes melanoma metastasis via altering the miRNA-539-3p/USP13/MITF/AXL signals. Oncogene.

[293] Rutkowski K., Sowa P., Rutkowska-Talipska J., Kuryliszyn-Moskal A., Rutkowski R. (2014). Dehydroepiandrosterone (DHEA): Hypes and hopes. Drugs.

[294] Teng Y., Litchfield L.M., Ivanova M.M., Prough R.A., Clark B.J., Klinge C.M. (2014). Dehydroepiandrosterone-induces miR-21 transcription in HepG2 cells through estrogen receptor β and androgen receptor. Mol. Cell. Endocrinol..

[295] Sustarsic E.G., Junnila R.K., Kopchick J.J. (2013). Human metastatic melanoma cell lines express high levels of growth hormone receptor and respond to GH treatment. Biochem. Biophys. Res. Commun..

[296] Basu R., Wu S., Kopchick J.J. (2017). Targeting growth hormone receptor in human melanoma cells attenuates tumor progression and epithelial mesenchymal transition via suppression of multiple oncogenic pathways. Oncotarget.

[297] Basu R., Kulkarni P., Qian Y., Walsh C., Arora P., Davis E., Duran-Ortiz S., Funk K., Ibarra D., Kruse C. (2019). Growth hormone upregulates melanocyte-inducing transcription factor expression and activity via JAK2-STAT5 and SRC signaling in GH receptor-positive human melanoma. Cancers.

[298] Palabiyik O., Tastekin E., Doganlar Z.B., Tayfur P., Dogan A., Vardar S.A. (2019). Alteration in cardiac PI3K/Akt/mTOR and ERK signaling pathways with the use of growth hormone and swimming, and the roles of miR21 and miR133. Biomed. Rep..

[299] Caldarola G., Battista C., Pellicano R. (2010). Melanoma onset after estrogen, thyroid, and growth hormone replacement therapy. Clin. Ther..

[300] Handler M.Z., Ross A.L., Shiman M.I., Elgart G.W., Grichnik J.M. (2012). Potential role of human growth hormone in melanoma growth promotion. Arch. Dermatol..

[301] Melnik B.C., John S.M., Schmitz G. (2013). Milk is not just food but most likely a genetic transfection system activating mTORC1 signaling for postnatal growth. Nutr. J..

[302] Rich-Edwards J.W., Ganmaa D., Pollak M.N., Nakamoto E.K., Kleinman K., Tserendolgor U., Willett W.C., Frazier A.L. (2007). Milk consumption and the prepubertal somatotropic axis. Nutr. J..

[303] Van Vught A.J., Nieuwenhuizen A.G., Veldhorst M.A., Brummer R.J., Westerterp-Plantenga M.S. (2010). The effects of dietary protein on the somatotropic axis: A comparison of soy, gelatin, alpha-lactalbumin and milk. Eur. J. Clin. Nutr..

[304] Melnik B.C. (2009). Androgen abuse in the community. Curr. Opin. Endocrinol. Diabetes Obes..

[305] Fearfield L., Nobbs J., Petruckevitch A., Harland C. (2019). Severe vitamin D deficiency associated with BRAF-mutated melanoma. Br. J. Dermatol..

[306] Newton-Bishop J.A., Beswick S., Randerson-Moor J., Chang Y.M., Affleck P., Elliott F., Chan M., Leake S., Karpavicius B., Haynes S. (2009). Serum 25-hydroxyvitamin D3 levels are associated with Breslow thickness at presentation and survival from melanoma. J. Clin. Oncol..

[307] Bade B., Zdebik A., Wagenpfeil S., Gräber S., Geisel J., Vogt T., Reichrath J. (2014). Low serum 25-hydroxyvitamin D concentrations are associated with increased risk for melanoma and unfavourable prognosis. PLoS ONE.

[308] Slominski A.T., Brożyna A.A., Zmijewski M.A., Jóźwicki W., Jetten A.M., Mason R.S., Tuckey R.C., Elmets C.A. (2017). Vitamin D signaling and melanoma: Role of vitamin D and its receptors in melanoma progression and management. Lab. Investig..

[309] Slominski A.T., Brożyna A.A., Skobowiat C., Zmijewski M.A., Kim T.K., Janjetovic Z., Oak A.S., Jozwicki W., Jetten A.M., Mason R.S. (2018). On the role of classical and novel forms of vitamin D in melanoma progression and management. J. Steroid Biochem. Mol. Biol..

[310] Stucci L.S., D′Oronzo S., Tucci M., Macerollo A., Ribero S., Spagnolo F., Marra E., Picasso V., Orgiano L., Marconcini R. (2018). Vitamin D in melanoma: Controversies and potential role in combination with immune check-point inhibitors. Cancer Treat. Rev..

[311] Dambal S., Giangreco A.A., Acosta A.M., Fairchild A., Richards Z., Deaton R., Wagner D., Vieth R., Gann P.H., Kajdacsy-Balla A. (2017). microRNAs and DICER1 are regulated by 1,25-dihydroxyvitamin D in prostate stroma. J. Steroid Biochem. Mol. Biol..

[312] Xu Y., Qian J., Yu Z. (2019). Budesonide up-regulates vitamin D receptor expression in human bronchial fibroblasts and enhances the inhibitory effect of calcitriol on airway remodeling. Allergol. Immunopathol. (Madr).

[313] Zou M., BinHumaid F.S., Alzahrani A.S., Baitei E.Y., Al-Mohanna F.A., Meyer B.F., Shi Y. (2014). Increased CYP24A1 expression is associated with BRAF(V600E) mutation and advanced stages in papillary thyroid carcinoma. Clin. Endocrinol. (Oxf.).

[314] Ohyama Y., Noshiro M., Okuda K. (1991). Cloning and expression of cDNA encoding 25-hydroxyvitamin D3 24-hydroxylase. FEBS Lett..

[315] Brożyna A.A., Jochymski C., Janjetovic Z., Jóźwicki W., Tuckey R.C., Slominski A.T. (2014). CYP24A1 expression inversely correlates with melanoma progression: Clinic-pathological studies. Int. J. Mol. Sci..

[316] Wacker M., Holick M.F. (2013). Sunlight and vitamin D: A global perspective for health. Dermatoendocrinol..

[317] Tuckey R.C., Li W., Ma D., Cheng C.Y.S., Wang K.M., Kim T.K., Jeayeng S., Slominski A.T. (2018). CYP27A1 acts on the pre-vitamin D3 photoproduct, lumisterol, producing biologically active hydroxy-metabolites. J. Steroid. Biochem. Mol. Biol..

[318] Slominski A.T., Kim T.K., Hobrath J.V., Janjetovic Z., Oak A.S.W., Postlethwaite A., Lin Z., Li W., Takeda Y., Jetten A.M. (2017). Characterization of a new pathway that activates lumisterol in vivo to biologically active hydroxylumisterols. Sci. Rep..

[319] Slominski A., Kim T.K., Zmijewski M.A., Janjetovic Z., Li W., Chen J., Kusniatsova E.I., Semak I., Postlethwaite A., Miller D.D. (2013). Novel vitamin D photoproducts and their precursors in the skin. Dermatoendocrinology.

[320] Merrill S.J., Ashrafi S., Subramanian M., Godar D.E. (2015). Exponentially increasing incidences of cutaneous malignant melanoma in Europe correlate with low personal annual UV doses and suggests 2 major risk factors. Dermatoendocrinology.

[321] Montero I., Requena C., Traves V., García-Casado Z., Kumar R., Nagore E. (2015). Age-related characteristics of cutaneous melanoma in a Spanish Mediterranean population. Int. J. Dermatol..

[322] Cavanaugh-Hussey M.W., Mu E.W., Kang S., Balch C.M., Wang T. (2015). Older age is associated with a higher incidence of melanoma death but a lower incidence of sentinel lymph node metastasis in the SEER databases (2003–2011). Ann. Surg. Oncol..

[323] Ribero S., Stucci L.S., Marra E., Marconcini R., Spagnolo F., Orgiano L., Picasso V., Queirolo P., Palmieri G., Quaglino P. (2018). Effect of age on melanoma risk, prognosis and treatment response. Acta Derm. Venereol..

[324] Olivieri F., Spazzafumo L., Santini G., Lazzarini R., Albertini M.C., Rippo M.R., Galeazzi R., Abbatecola A.M., Marcheselli F., Monti D. (2012). Age-related differences in the expression of circulating microRNAs: miR-21 as a new circulating marker of inflammaging. Mech. Ageing Dev..

[325] Halper B., Hofmann M., Oesen S., Franzke B., Stuparits P., Vidotto C., Tschan H., Bachl N., Strasser E.M., Quittan M. (2015). Influence of age and physical fitness on miRNA-21, TGF-β and its receptors in leukocytes of healthy women. Exerc. Immunol. Rev..

[326] Zitvogel L., Pietrocola F., Kroemer G. (2017). Nutrition, inflammation and cancer. Nat. Immunol..

[327] Yu H., Pardoll D., Jove R. (2009). STATs in cancer inflammation and immunity: A leading role for STAT3. Nat. Rev. Cancer.

[328] Fan Y., Mao R., Yang J. (2013). NF-κB and STAT3 signaling pathways collaboratively link inflammation to cancer. Protein Cell..

[329] Xue Z., Xi Q., Liu H., Guo X., Zhang J., Zhang Z., Li Y., Yang G., Zhou D., Yang H. (2019). miR-21 promotes NLRP3 inflammasome activation to mediate pyroptosis and endotoxic shock. Cell Death Dis.

[330] Ning Z.W., Luo X.Y., Wang G.Z., Li Y., Pan M.X., Yang R.Q., Ling X.G., Huang S., Ma X.X., Jin S.Y. (2017). MicroRNA-21 mediates angiotensin II-induced liver fibrosis by activating NLRP3 inflammasome/IL-1β axis via targeting Smad7 and Spry1. Antioxid. Redox Signal..

[331] Loboda A., Sobczak M., Jozkowicz A., Dulak J. (2016). TGF-β1/Smads and miR-21 in renal fibrosis and inflammation. Mediators Inflamm..

[332] Specjalski K., Jassem E. (2019). MicroRNAs: Potential biomarkers and targets of therapy in allergic diseases?. Arch. Immunol. Ther. Exp. (Warsz.).

[333] Van den Berge M., Tasena H. (2019). Role of microRNAs and exosomes in asthma. Curr. Opin. Pulm. Med..

[334] Isanejad A., Alizadeh A.M., Amani Shalamzari S., Khodayari H., Khodayari S., Khori V., Khojastehnjad N. (2016). MicroRNA-206, let-7a and microRNA-21 pathways involved in the anti-angiogenesis effects of the interval exercise training and hormone therapy in breast cancer. Life Sci..

[335] Nielsen S., Åkerström T., Rinnov A., Yfanti C., Scheele C., Pedersen B.K., Laye M.J. (2014). The miRNA plasma signature in response to acute aerobic exercise and endurance training. PLoS ONE.

[336] Pinto R., Strippoli S., De Summa S., Albano A., Azzariti A., Guida G., Popescu O., Lorusso V., Guida M., Tommasi S. (2015). MicroRNA expression in BRAF-mutated and wild-type metastatic melanoma and its correlation with response duration to BRAF inhibitors. Expert Opin. Ther. Targets.

[337] Lunavat T.R., Cheng L., Einarsdottir B.O., Olofsson Bagge R., Veppil Muralidharan S., Sharples R.A., Lässer C., Gho Y.S., Hill A.F., Nilsson J.A. (2017). BRAFV600 inhibition alters the microRNA cargo in the vesicular secretome of malignant melanoma cells. Proc. Natl. Acad. Sci. USA.

[338] Jaune E., Rocchi S. (2018). Metformin: Focus on melanoma. Front. Endocrinol..

[339] De Souza Neto F.P., Bernardes S.S., Marinello P.C., Melo G.P., Luiz R.C., Cecchini R., Cecchini A. (2017). Metformin: Oxidative and proliferative parameters in-vitro and in-vivo models of murine melanoma. Melanoma Res..

[340] Li K., Zhang T.T., Wang F., Cui B., Zhao C.X., Yu J.J., Lv X.X., Zhang X.W., Yang Z.N., Huang B. (2018). Metformin suppresses melanoma progression by inhibiting KAT5-mediated SMAD3 acetylation, transcriptional activity and TRIB3 expression. Oncogene.

[341] Ryabaya O., Prokofieva A., Akasov R., Khochenkov D., Emelyanova M., Burov S., Markvicheva E., Inshakov A., Stepanova E. (2019). Metformin increases antitumor activity of MEK inhibitor binimetinib in 2D and 3D models of human metastatic melanoma cells. Biomed. Pharmacother..

[342] Montaudié H., Cerezo M., Bahadoran P., Roger C., Passeron T., Machet L., Arnault J.P., Verneuil L., Maubec E., Aubin F. (2017). Metformin monotherapy in melanoma: A pilot, open-label, prospective, and multicentric study indicates no benefit. Pigment Cell Melanoma Res..

[343] Vujic I., Sanlorenzo M., Posch C., Esteve-Puig R., Yen A.J., Kwong A., Tsumura A., Murphy R., Rappersberger K., Ortiz-Urda S. (2015). Metformin and trametinib have synergistic effects on cell viability and tumor growth in NRAS mutant cancer. Oncotarget.

[344] Afzal M.Z., Mercado R.R., Shirai K. (2018). Efficacy of metformin in combination with immune checkpoint inhibitors (anti-PD-1/anti-CTLA-4) in metastatic malignant melanoma. J. Immunother. Cancer.

[345] Tseng H.W., Li S.C., Tsai K.W. (2019). Metformin treatment suppresses melanoma cell growth and motility through modulation of microRNA expression. Cancers.

[346] Hirsch H.A., Iliopoulos D., Struhl K. (2013). Metformin inhibits the inflammatory response associated with cellular transformation and cancer stem cell growth. Proc. Natl. Acad. Sci. USA.

[347] Leidgens V., Proske J., Rauer L., Moeckel S., Renner K., Bogdahn U., Riemenschneider M.J., Proescholdt M., Vollmann-Zwerenz A., Hau P. (2017). Stattic and metformin inhibit brain tumor initiating cells by reducing STAT3-phosphorylation. Oncotarget.

[348] Esparza-López J., Alvarado-Muñoz J.F., Escobar-Arriaga E., Ulloa-Aguirre A., de Jesús Ibarra-Sánchez M. (2019). Metformin reverses mesenchymal phenotype of primary breast cancer cells through STAT3/NF-κB pathways. BMC Cancer.

[349] Wang J., Gao Y., Duan L., Wei S., Liu J., Tian L., Quan J., Zhang Q., Liu J., Yang J. (2017). Metformin ameliorates skeletal muscle insulin resistance by inhibiting miR-21 expression in a high-fat dietary rat model. Oncotarget.

[350] Luo M., Tan X., Mu L., Luo Y., Li R., Deng X., Chen N., Ren M., Li Y., Wang L. (2017). MiRNA-21 mediates the antiangiogenic activity of metformin through targeting PTEN and SMAD7 expression and PI3K/AKT pathway. Sci. Rep..

[351] Deng Y., Ma W. (2018). Metformin inhibits HaCaT cell viability via the miR-21/PTEN/Akt signaling pathway. Mol. Med. Rep..

[352] Demirsoy İ.H., Ertural D.Y., Balci Ş., Çınkır Ü., Sezer K., Tamer L., Aras N. (2018). Profiles of circulating miRNAs following metformin treatment in patients with type 2 diabetes. J. Med. Biochem..

[353] Bao B., Azmi A.S., Ali S., Zaiem F., Sarkar F.H. (2014). Metformin may function as anti-cancer agent via targeting cancer stem cells: The potential biological significance of tumor-associated miRNAs in breast and pancreatic cancers. Ann. Transl. Med..

[354] Kokolus K.M., Zhang Y., Sivik J.M., Schmeck C., Zhu J., Repasky E.A., Drabick J.J., Schell T.D. (2017). Beta blocker use correlates with better overall survival in metastatic melanoma patients and improves the efficacy of immunotherapies in mice. Oncoimmunology.

[355] De Giorgi V., Grazzini M., Benemei S., Marchionni N., Botteri E., Pennacchioli E., Geppetti P., Gandini S. (2018). Propranolol for off-label treatment of patients with melanoma: Results from a cohort study. JAMA Oncol..

[356] Bustamante P., Miyamoto D., Goyeneche A., de Alba Graue P.G., Jin E., Tsering T., Dias A.B., Burnier M.N., Burnier J.V. (2019). Beta-blockers exert potent anti-tumor effects in cutaneous and uveal melanoma. Cancer Med..

[357] Sayed D., Rane S., Lypowy J., He M., Chen I.Y., Vashistha H., Yan L., Malhotra A., Vatner D., Abdellatif M. (2008). MicroRNA-21 targets Sprouty2 and promotes cellular outgrowths. Mol. Biol. Cell.

[358] Hou Y., Sun Y., Shan H., Li X., Zhang M., Zhou X., Xing S., Sun H., Chu W., Qiao G. (2012). β-adrenoceptor regulates miRNA expression in rat heart. Med. Sci. Monit..

[359] Liu D., Yang Z., Wang T., Yang Z., Chen H., Hu Y., Hu C., Guo L., Deng Q., Liu Y. (2016). β2-AR signaling controls trastuzumab resistance-dependent pathway. Oncogene.

[360] Gryshkova V., Fleming A., McGhan P., De Ron P., Fleurance R., Valentin J.P., Nogueira da Costa A. (2018). miR-21-5p as a potential biomarker of inflammatory infiltration in the heart upon acute drug-induced cardiac injury in rats. Toxicol. Lett..

[361] Carrillo E.D., Escobar Y., González G., Hernández A., Galindo J.M., García M.C., Sánchez J.A. (2011). Posttranscriptional regulation of the β2-subunit of cardiac L-type Ca2+ channels by MicroRNAs during long-term exposure to isoproterenol in rats. J. Cardiovasc. Pharmacol..

[362] Zhang W., Qu X., Chen B., Snyder M., Wang M., Li B., Tang Y., Chen H., Zhu W., Zhan L. (2016). Critical roles of STAT3 in β-adrenergic functions in the heart. Circulation.

[363] Balligand J.L. (2016). β-Adrenergic receptors cooperate with transcription factors: The “STAT” of their union. Circulation.

[364] Chimenti I., Pagano F., Cavarretta E., Angelini F., Peruzzi M., Barretta A., Greco E., De Falco E., Marullo A.G., Sciarretta S. (2016). Β-blockers treatment of cardiac surgery patients enhances isolation and improves phenotype of cardiosphere-derived cells. Sci. Rep..

[365] Ji Y., Chen S., Xu C., Li L., Xiang B. (2015). The use of propranolol in the treatment of infantile haemangiomas: An update on potential mechanisms of action. Br. J. Dermatol..

[366] Miroshnichenko S.K., Patutina O.A., Burakova E.A., Chelobanov B.P., Fokina A.A., Vlassov V.V., Altman S., Zenkova M.A., Stetsenko D.A. (2019). Mesyl phosphoramidate antisense oligonucleotides as an alternative to phosphorothioates with improved biochemical and biological properties. Proc. Natl. Acad. Sci. USA.

[367] Patutina O.A., Miroshnichenko S.K., Mironova N.L., Sen′kova A.V., Bichenkova E.V., Clarke D.J., Vlassov V.V., Zenkova M.A. (2019). Catalytic knockdown of miR-21 by artificial ribonuclease: Biological performance in tumor model. Front. Pharmacol..

[368] Bhere D., Arghiani N., Lechtich E.R., Yao Y., Alsaab S., Bei F., Matin M.M., Shah K. (2020). Simultaneous downregulation of miR-21 and upregulation of miR-7 has anti-tumor efficacy. Sci. Rep..

[369] Wei X., You X., Zhang J., Zhou C. (2019). miR-21 inhibitor facilitates the anticancer activity of doxorubicin loaded nanometer in melanoma. Oncol. Rep..

[370] Zhang H.L., Si L.B., Zeng A., Long F., Qi Z., Zhao R., Bai M. (2018). MicroRNA-21 antisense oligonucleotide improves the sensitivity of A375 human melanoma cell to Cisplatin: An in vitro study. J. Cell. Biochem..

[371] Rui M., Qu Y., Gao T., Ge Y., Feng C., Xu X. (2016). Simultaneous delivery of anti-miR21 with doxorubicin prodrug by mimetic lipoprotein nanoparticles for synergistic effect against drug resistance in cancer cells. Int. J. Nanomed..

[372] Liang G., Zhu Y., Ali D.J., Tian T., Xu H., Si K., Sun B., Chen B., Xiao Z. (2020). Engineered exosomes for targeted co-delivery of miR-21 inhibitor and chemotherapeutics to reverse drug resistance in colon cancer. J. Nanobiotechnol..

[373] Agha A., Tarhini A.A. (2017). Adjuvant therapy for melanoma. Curr. Oncol. Rep..

[374] Bazhin A.V., von Ahn K., Fritz J., Werner J., Karakhanova S. (2018). Interferon-α up-regulates the expression of PD-L1 molecules on immune cells through STAT3 and p38 signaling. Front. Immunol..

[375] Reinsbach S., Nazarov P.V., Philippidou D., Schmitt M., Wienecke-Baldacchino A., Muller A., Vallar L., Behrmann I., Kreis S. (2012). Dynamic regulation of microRNA expression following interferon-γ-induced gene transcription. RNA Biol..

[376] Li H., Yuan S.M., Yang M., Zha H., Li X.R., Sun H., Duan L., Gu Y., Li A.F., Weng Y.G. (2016). High intensity focused ultrasound inhibits melanoma cell migration and metastasis through attenuating microRNA-21-mediated PTEN suppression. Oncotarget.

[377] Labala S., Jose A., Chawla S.R., Khan M.S., Bhatnagar S., Kulkarni O.P., Venuganti V.V.K. (2017). Effective melanoma cancer suppression by iontophoretic co-delivery of STAT3 siRNA and imatinib using gold nanoparticles. Int. J. Pharm..

[378] Zhou X., Yuan P., Liu Q., Liu Z. (2017). LncRNA MEG3 regulates imatinib resistance in chronic myeloid leukemia via suppressing microRNA-21. Biomol. Ther. (Seoul).

[379] Nabavi S.M., Russo G.L., Tedesco I., Daglia M., Orhan I.E., Nabavi S.F., Bishayee A., Nagulapalli Venkata K.C., Abdollahi M., Hajheydari Z. (2018). Curcumin and melanoma: From chemistry to medicine. Nutr. Cancer.

[380] Yang C.H., Yue J., Sims M., Pfeffer L.M. (2013). The curcumin analog EF24 targets NF-κB and miRNA-21, and has potent anticancer activity in vitro and in vivo. PLoS ONE.

[381] Lelli D., Pedone C., Sahebkar A. (2017). Curcumin and treatment of melanoma: The potential role of microRNAs. Biomed. Pharmacother..

[382] Mudduluru G., George-William J.N., Muppala S., Asangani I.A., Kumarswamy R., Nelson L.D., Allgayer H. (2011). Curcumin regulates miR-21 expression and inhibits invasion and metastasis in colorectal cancer. Biosci. Rep..

[383] Chen J., Xu T., Chen C. (2015). The critical roles of miR-21 in anti-cancer effects of curcumin. Ann. Transl. Med..

[384] Tahata S., Singh S.V., Lin Y., Hahm E.R., Beumer J.H., Christner S.M., Rao U.N., Sander C., Tarhini A.A., Tawbi H. (2018). Evaluation of biodistribution of sulforaphane after administration of oral broccoli sprout extract in melanoma patients with multiple atypical nevi. Cancer Prev. Res..

[385] Arcidiacono P., Ragonese F., Stabile A., Pistilli A., Kuligina E., Rende M., Bottoni U., Calvieri S., Crisanti A., Spaccapelo R. (2018). Antitumor activity and expression profiles of genes induced by sulforaphane in human melanoma cells. Eur. J. Nutr..

[386] Rudolf K., Cervinka M., Rudolf E. (2014). Sulforaphane-induced apoptosis involves p53 and p38 in melanoma cells. Apoptosis.

[387] Mitsiogianni M., Koutsidis G., Mavroudis N., Trafalis D.T., Botaitis S., Franco R., Zoumpourlis V., Amery T., Galanis A., Pappa A. (2019). The role of isothiocyanates as cancer chemo-preventive, chemo-therapeutic and anti-melanoma agents. Antioxidants.

[388] Martin S.L., Kala R., Tollefsbol T.O. (2018). Mechanisms for the inhibition of colon cancer cells by sulforaphane through epigenetic modulation of microRNA-21 and human telomerase reverse transcriptase (hTERT) down-regulation. Curr. Cancer Drug Targets.

[389] Lan F., Pan Q., Yu H., Yue X. (2015). Sulforaphane enhances temozolomide-induced apoptosis because of down-regulation of miR-21 via Wnt/β-catenin signaling in glioblastoma. J. Neurochem..

[390] Shen Q., Tian F., Jiang P., Li Y., Zhang L., Lu J., Li J. (2009). EGCG enhances TRAIL-mediated apoptosis in human melanoma A375 cell line. J. Huazhong Univ. Sci. Technolog. Med. Sci..

[391] Ellis L.Z., Liu W., Luo Y., Okamoto M., Qu D., Dunn J.H., Fujita M. (2011). Green tea polyphenol epigallocatechin-3-gallate suppresses melanoma growth by inhibiting inflammasome and IL-1β secretion. Biochem. Biophys. Res. Commun..

[392] Zhang J., Lei Z., Huang Z., Zhang X., Zhou Y., Luo Z., Zeng W., Su J., Peng C., Chen X. (2016). Epigallocatechin-3-gallate (EGCG) suppresses melanoma cell growth and metastasis by targeting TRAF6 activity. Oncotarget.

[393] Fujiki H., Watanabe T., Sueoka E., Rawangkan A., Suganuma M. (2018). Cancer prevention with green tea and its principal constituent, EGCG: From early investigations to current focus on human cancer stem cells. Mol. Cells.

[394] Chen X., Chang L., Qu Y., Liang J., Jin W., Xia X. (2018). Tea polyphenols inhibit the proliferation, migration, and invasion of melanoma cells through the down-regulation of TLR4. Int. J. Immunopathol. Pharmacol..

[395] Khoi P.N., Park J.S., Kim J.H., Xia Y., Kim N.H., Kim K.K., Jung Y.D. (2013). (-)-Epigallocatechin-3-gallate blocks nicotine-induced matrix metalloproteinase-9 expression and invasiveness via suppression of NF-κB and AP-1 in endothelial cells. Int. J. Oncol..

[396] Kim J.E., Shin M.H., Chung J.H. (2013). Epigallocatechin-3-gallate prevents heat shock-induced MMP-1 expression by inhibiting AP-1 activity in human dermal fibroblasts. Arch. Dermatol. Res..

[397] Siddiqui I.A., Asim M., Hafeez B.B., Adhami V.M., Tarapore R.S., Mukhtar H. (2011). Green tea polyphenol EGCG blunts androgen receptor function in prostate cancer. FASEB J..

[398] Fix L.N., Shah M., Efferth T., Farwell M.A., Zhang B. (2010). MicroRNA expression profile of MCF-7 human breast cancer cells and the effect of green tea polyphenon-60. Cancer Genom. Proteom..

[399] Schlumpf M., Reichrath J., Lehmann B., Sigmundsdottir H., Feldmeyer L., Hofbauer G.F., Lichtensteiger W. (2010). Fundamental questions to sun protection: A continuous education symposium on vitamin D, immune system and sun protection at the University of Zürich. Dermatoendocrinology.

[400] Reichrath J., Reichrath S. (2014). Sunlight, vitamin D and malignant melanoma: An update. Adv. Exp. Med. Biol..

[401] Huerter C.J., Vaudreuil A., Agrawal D.K., Nguyen A.H. (2016). Has vitamin D had its “time in the sun” for melanoma?. J. Clin. Aesthet. Dermatol..

[402] De Smedt J., Van Kelst S., Boecxstaens V., Stas M., Bogaerts K., Vanderschueren D., Aura C., Vandenberghe K., Lambrechts D., Wolter P. (2017). Vitamin D supplementation in cutaneous malignant melanoma outcome (ViDMe): A randomized controlled trial. BMC Cancer.

[403] Brożyna A.A., Hoffman R.M., Slominski A.T. (2020). Relevance of vitamin D in melanoma development, progression and therapy. Anticancer Res..

[404] Moore S.C., Lee I.M., Weiderpass E., Campbell P.T., Sampson J.N., Kitahara C.M., Keadle S.K., Arem H., Berrington de Gonzalez A., Hartge P. (2016). Association of leisure-time physical activity with risk of 26 types of cancer in 1.44 million adults. JAMA Intern. Med..

[405] Shors A.R., Solomon C., McTiernan A., White E. (2001). Melanoma risk in relation to height, weight, and exercise (United States). Cancer Causes Control.

[406] Gogas H., Trakatelli M., Dessypris N., Terzidis A., Katsambas A., Chrousos G.P., Petridou E.T. (2008). Melanoma risk in association with serum leptin levels and lifestyle parameters: A case-control study. Ann. Oncol..

[407] Ruiz-Casado A., Martín-Ruiz A., Pérez L.M., Provencio M., Fiuza-Luces C., Lucia A. (2017). Exercise and the hallmarks of cancer. Trends Cancer.

[408] Cancer Genome Atlas Network (2015). Genomic classification of cutaneous melanoma. Cell.

[409] Zhang T., Dutton-Regester K., Brown K.M., Haywar N.K. (2016). The genomic landscape of cutaneous melanoma. Pigment Cell Melanoma Res..

[410] Hayward N.K., Wilmott J.S., Waddell N., Johansson P.A., Field M.A., Nones K., Patch A.M., Kakavand H., Alexandrov L.B., Burke H. (2017). Whole-genome landscapes of major melanoma subtypes. Nature.

[411] Shain A.H., Joseph N.M., Yu R., Benhamida J., Liu S., Prow T., Ruben B., North J., Pincus L., Yeh I. (2018). Genomic and transcriptomic analysis reveals incremental disruption of key signaling pathways during melanoma evolution. Cancer Cell.

[412] Moon H., Donahue L.R., Choi E., Scumpia P.O., Lowry W.E., Grenier J.K., Zhu J., White A.C. (2017). Melanocyte stem cell activation and translocation initiate cutaneous melanoma in response to UV exposure. Cell Stem Cell.

[413] Lorusso C., De Summa S., Pinto R., Danza K., Tommas S. (2020). miRNAs as key players in the management of cutaneous melanoma. Cells.

